# A Cell/Cilia Cycle Biosensor for Single-Cell Kinetics Reveals Persistence of Cilia after G1/S Transition Is a General Property in Cells and Mice

**DOI:** 10.1016/j.devcel.2018.10.027

**Published:** 2018-11-19

**Authors:** Matthew J. Ford, Patricia L. Yeyati, Girish R. Mali, Margaret A. Keighren, Scott H. Waddell, Heidi K. Mjoseng, Adam T. Douglas, Emma A. Hall, Asako Sakaue-Sawano, Atsushi Miyawaki, Richard R. Meehan, Luke Boulter, Ian J. Jackson, Pleasantine Mill, Richard L. Mort

**Affiliations:** 1 MRC Human Genetics Unit, MRC Institute of Genetics & Molecular Medicine, University of Edinburgh, Western General Hospital, Edinburgh EH4 2XU, UK; 2 Centre of Brain Science, Laboratory for Cell Function and Dynamics, RIKEN, 2-1 Hirosawa, Wako, Saitama 351-0198, Japan; 3 Roslin Institute, University of Edinburgh, Roslin EH25 9RG, UK; 4 Division of Biomedical and Life Sciences, Faculty of Health and Medicine, Lancaster University, Bailrigg, Furness Building, Lancaster LA1 4YG, UK

**Keywords:** cilia, cell cycle, reporter mouse, *Rosa26*, live imaging, biosensor, organoid

## Abstract

The cilia and cell cycles are inextricably linked. Centrioles in the basal body of cilia nucleate the ciliary axoneme and sequester pericentriolar matrix (PCM) at the centrosome to organize the mitotic spindle. Cilia themselves respond to growth signals, prompting cilia resorption and cell cycle re-entry. We describe a fluorescent cilia and cell cycle biosensor allowing live imaging of cell cycle progression and cilia assembly and disassembly kinetics in cells and inducible mice. We define assembly and disassembly in relation to cell cycle stage with single-cell resolution and explore the intercellular heterogeneity in cilia kinetics. In all cells and tissues analyzed, we observed cilia that persist through the G1/S transition and into S/G2/M-phase. We conclude that persistence of cilia after the G1/S transition is a general property. This resource will shed light at an individual cell level on the interplay between the cilia and cell cycles in development, regeneration, and disease.

## Highlights

• Arl13bCerulean-Fucci2a biosensor labels the cell and cilia cycles
• Analysis of cells and mice reveals persistence of cilia after the G1/S transition
• Inducible mouse line allows lineage tracing and *ex vivo* live imaging
• Organisms can tolerate artificially lengthened cilia without overt phenotypes.

## Graphical Abstract

**Figure:**
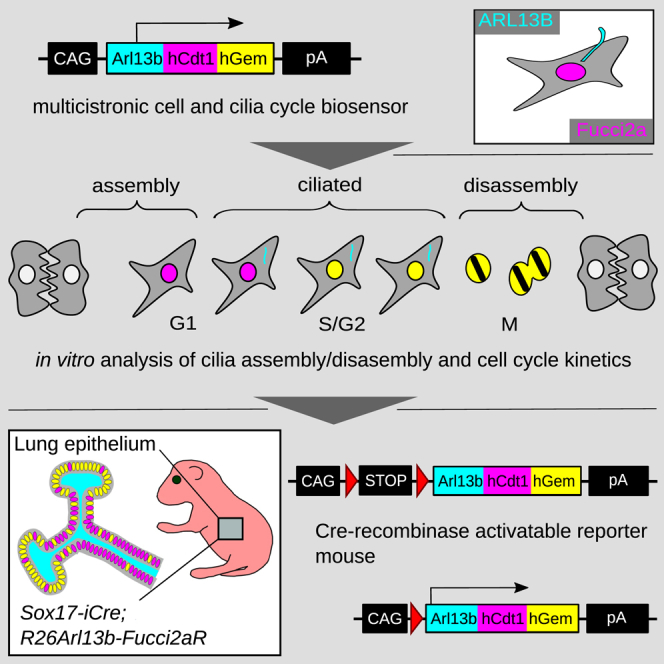

## Teaser

The cilia and cell cycles are fundamental processes coupled through shared machinery. Ford et al. develop and characterize a multicistronic biosensor that can simultaneously label the cell and cilia cycles in mice, enabling live imaging studies of their kinetics.

## Introduction

Cilia are microtubule-based cellular projections that come in motile and non-motile forms. They sense key mechanical and environmental cues including the transduction of mitogenic signals that include Hedgehog (HH), insulin-like growth factor 1 (IGF-1), and platelet-derived growth factor (PDGF) (Goetz and Anderson, 2010). Defects in cilia result in a broad group of disorders termed ciliopathies. The structural and functional diversity of mammalian cilia likely underlies the huge spectrum of phenotypes observed in ciliopathy patients (Reiter and Leroux, 2017). We currently know very little about cell type-specific and developmental stage-specific differences in cilia dynamics and their interplay with the cell cycle during tissue morphogenesis and disease.

Primary cilia are dynamic organelles whose assembly and resorption are inextricably coupled with cell cycle progression. Evidence connecting primary cilia and the cell cycle, from mostly cell culture studies, suggests that primary cilia may function as a structural checkpoint guarding against cell cycle re-entry (Izawa et al., 2015). The spatially distinct dual functions of the centrioles as structural components of both the basal body of the cilium and centrosomes at the poles of the mitotic spindle likely contribute to this coordination. The basal body must be disassembled prior to mitosis in order for the spindle to form and then differentiate in the subsequent cell cycle, enabling it to dock to the plasma membrane and form the ciliary axoneme (reviewed in Seeley and Nachury, 2010). Evidence also exists for extensive molecular crosstalk between ciliary factors and key cell cycle regulators; for example, the anaphase promoting complex (APC) may be sequestered at the cilium and localized cell cycle-dependent proteolysis of the CDK5-SCF-Nde1 axis may trigger cell cycle progression (Maskey et al., 2015, Pugacheva et al., 2007, Wang et al., 2014).

In disease, the interplay between the cilia and cell cycles is complex. The presence of cilia is antiproliferative in some cases of cancer and polycystic kidney disease (Lin et al., 2003, Jonassen et al., 2008, Hassounah et al., 2013, Menzl et al., 2014, Phua et al., 2017). Conversely, proproliferative ciliary-dependent signaling through the HH and Wnt pathways can drive the development of cancer (Han et al., 2009, Ma et al., 2013, Wong et al., 2009). Furthermore, there are causative links between the mis-regulation of cilia length and stability and kinase inhibitor resistance in cancer (Jenks et al., 2018). These examples highlight the translational importance of better understanding cilia dynamics and signaling in cell type- and disease-specific contexts.

Clearly, strategies for parallel analysis of cell cycle progression and ciliary parameters *in vivo* are required to advance many fields. The fluorescent ubiquitination-based cell cycle indicator (Fucci2) system consists of cell cycle biosensors incorporating truncated forms of the human cell cycle phase-specific proteins CDT1 (amino acids 30–120) and Geminin (amino acids 1–110) and the fluorescent proteins mCherry and mVenus, respectively (Sakaue-Sawano et al., 2008, Abe et al., 2013). *R26Fucci2aR* is a Cre-inducible cell cycle reporter mouse that incorporates the Fucci2 probes fused with the *Thosea asigna* virus 2A self-cleaving peptide sequence in a single bicistronic construct (Mort et al., 2014).

The ciliary protein ARL13B is a small GTPase enriched in cilia and is required for cilium assembly and HH signaling (Caspary et al., 2007). *ARL13B* mutations are causal in a subset of patients with classical Joubert syndrome (JS, MIM:608922), characterized by an abnormal MRI, ataxia, psychomotor delay, and cerebellar vermis hypoplasia. Overexpression of wild-type human ARL13B is tolerated in zebrafish and can rescue the JS-like phenotype in *arl13b*^*sco*^ mutants (Cantagrel et al., 2008). Several transgenic models exist that label primary and motile cilia by fusion of wild-type ARL13B to fluorescent proteins (Borovina et al., 2010, Delling et al., 2013, Bangs et al., 2015, Schmitz et al., 2017). These models exhibit no adverse gross phenotypes; Shh signaling is not affected, embryos and tissues develop normally, and animals are healthy. However, consistent with ARL13B’s function in extending the ciliary axoneme and membrane, increased ciliary length has been reported upon ARL13B overexpression (Larkins et al., 2011, Lu et al., 2015, Pintado et al., 2015). Furthermore, abnormal periciliary mislocalization has been observed on ARL-13::GFP overexpression in *C. elegans* (Warburton-Pitt et al., 2014).

Here, we report the design, construction, and validation of tricistronic cilia and cell cycle biosensor incorporating ARL13B-Cerulean and Fucci2a and the development of a Cre-inducible *R26Arl13b-Fucci2aR* reporter mouse. The model we have generated allows capture of high-resolution images enabling identification of the cell cycle stage and ciliation state of individual cells in culture and in all tissues examined, both embryonic and adult. The *R26Arl13b-Fucci2aR* mouse is a powerful tool for the understanding of cilia and cell cycle kinetics during mouse development and disease progression.

### Design

#### An Arl13bCerulean-Fucci2a Tricistronic Biosensor Designed to Label the Cilia and Cell Cycles

**Figure 1:**
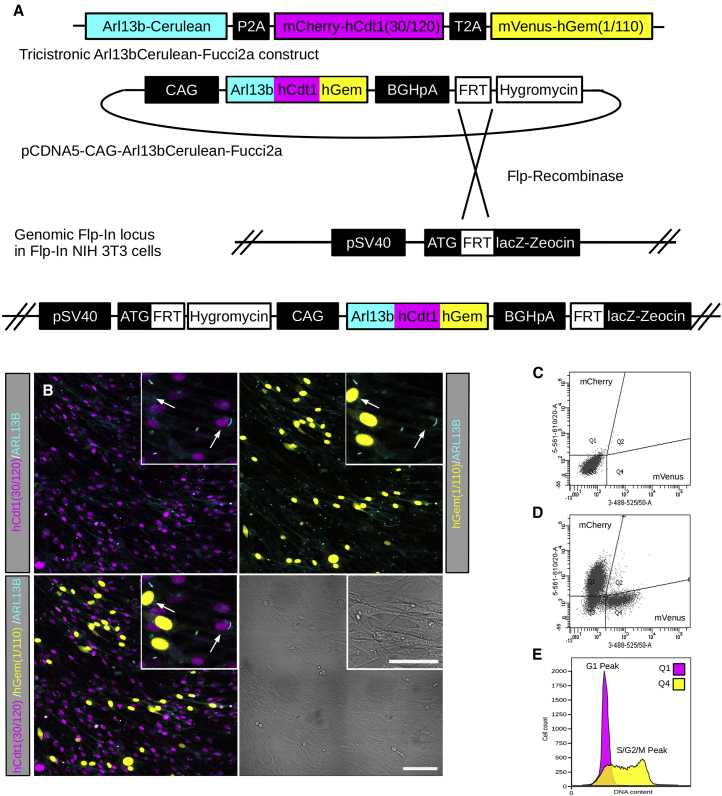
Design and Characterization of an Arl13bCerulean-Fucci2a Reporter with Stable Integration and Expression in an NIH 3T3 Cell Line (A) The full length mouse *Arl13b* cDNA was fused to mCerulean and combined with the Fucci2a probes mCherry-hCdt1(30/120) and mVenus-hGem(1/110) separated by the self-cleaving peptides P2A and T2A, respectively. Expression of this tricistronic construct is driven by the CAG promoter. A stable NIH 3T3 cell line was generated using the Flp-In system incorporating a single copy of Arl13bCerulean-Fucci2a by co-transfection of pCDNA5-CAG-Arl13bCerulean-Fucci2a and the Flp-recombinase expressing plasmid pOG44. (B) Live confocal images of the Arl13bCerulean-Fucci2a 3T3 cells showing nuclei distributed throughout the G1 or S/G2M cell cycle phases and labeled with mCherry-hCdt1(30/120) or mVenus-hGem(1/110), respectively. Single primary cilia are apparent on cells in both G1 and S/G2/M phases of the cell cycle (arrows in B, inset). (C and D) FACS analysis of Arl13bCerulean-Fucci2a 3T3 cells showed distinct mCherry-hCdt1(30/120) and mVenus-hGem(1/110) labeled cell populations (D) when compared to a control cell line (C). (E) DAPI staining and FACS analysis to determine the DNA content of the mCherry-hCdt1(30/120) and mVenus-hGem(1/110) populations in (C) confirmed faithful reporting of cell cycle stage; mCherry-hCdt1(30/120) positive cells exhibit a classical 2n peak confirming they are in the G1 cell cycle phase; mVenus-hGem(1/110) positive cells exhibit a long peak between 2n and 4n, confirming a population of cells in S, G2, and M phases of the cell cycle. Scale bars: 100 μm in (B) and 50 μm in (B) (inset).

In order to design a multicistronic construct to visualize cilia and cell cycle kinetics that could be expressed from a single locus in mice, we took advantage of the optimal spectral separation of mCerulean from the fluorescent components of the Fucci2a system, mCherry and mVenus (Rizzo et al., 2004, Shaner et al., 2005). We fused the full mouse *Arl13b* cDNA (EBI: OTTMUST00000058920.GRCm38) to mCerulean and coupled this construct to Fucci2a (Figure 1A), with expression driven by the CAG synthetic promoter (Miyazaki et al., 1989). To confirm that the construct was bright enough in a single copy number, we tested it in NIH 3T3 cells by incorporating a single copy of CAG-Arl13bCerulean-Fucci2a using the Flp/In system (Thermo Fisher Scientific, Massachusetts, USA) to generate a stable isogenic cell line (Figure 1A). We observed normal cell cycle progression and the correct localization of the Fucci2a probes to the nucleus (Figure 1B). mCherry-hCdt1(30/120) and mVenus-hGem(1/110) fluorescence correlated with the G1 and S/G2/M cell cycle phases, respectively, in our time-lapse sequences (Video S1, left panel). Cell cycle phase specificity of the Fucci2a probes was further confirmed by DNA content quantification using fluorescence-activated cell sorting (FACS) (Figures 1C–1E). In parallel, we observed primary cilia clearly labeled by ARL13B-Cerulean during cell cycle progression and as cells became confluent (Figure 1B; Video S1). The proportion of ciliated cells varied with the proportion of cells in the Fucci2a labeled cell cycle phases. Typically, in subconfluent cultures, 8.8% ± 2.87% of cells in G1 (n = 165 cells) were ciliated while in S/G2/M 11.0% ± 3.82% of cells (n = 119 cells) were ciliated. In confluent cultures, 66.1% ± 4.47% (n = 256 cells) and 73.0% ± 15.58% of cells (n = 23 cells) were ciliated in G1 and S/G2/M phases, respectively. We could resolve cilia at all stages of the cell cycle. In confluent cultures, cilia ranged in length from 2.52 μm to 21.38 μm; the mean (± 95% CI) length was 7.07 ± 0.33 μm (n = 221 cells). These data show that Arl13bCerulean-Fucci2a is bright enough as a single copy number insertion for us to resolve both the cell cycle stage-specific abundance of the Fucci2a probes and the localization of ARL13B-Cerulean to the primary cilia using live confocal microscopy.

Video S1. Primary Cilia are Apparent on Arl13bCerulean-Fucci2a NIH 3T3 Cells in Both G1 and S/G2/M Phases of the Cell Cycle, Related to Figure 1 Left panel: stable Arl13bCerulean-Fucci2a NIH 3T3 cells progressing through the cell and cilia cycles. Right panel: a single Arl13bCerulean-Fucci2a NIH 3T3 cell (arrow in frame 1) progresses through the cell cycle and divides to form two ciliated daughter cells (arrows in final frame). The cilia in the mother persist until late G2.

#### Generation of *R26Ar13b-Fucci2aR* Inducible Cell and Cilia Cycle Reporter Mice

*R26Arl13b-Fucci2aR* mice were generated by targeting a Cre-recombinase inducible version of Arl13bCerulean-Fucci2a to the *Rosa26* locus via homologous recombination in mESCs (Soriano, 1999) (Figures S1A–S1D; STAR Methods). Upon Cre-activation (see STAR Methods), we were able to identify the cell cycle phase by differential abundance of the Fucci2a probes and primary cilia labeled with ARL13B-Cerulean under 2i culture conditions (Figure S1D) in agreement with previous reports (Bangs et al., 2015). *R26Arl13b-Fucci2aR* mice were generated by blastocyst injection of correctly targeted *R26Arl13b-Fucci2aR* ES cells (MGI: 6193732). No expression of the transgene was detected by fluorescent imaging, confirming the functionality of the neomycin stop cassette (data not shown). To screen for abnormal phenotypes, a constitutive mouse line (*R26Arl13b-Fucci2a*, MGI: 6193734,) was generated by crossing with ubiquitous *CAG-Cre* animals (a gift from D.A. Kleinjan, University of Edinburgh) to delete the floxed neomycin stop cassette. In a cross to breed away the Cre-transgene *R26Arl13b-Fucci2a* mice were born at Mendelian ratios (n = 15, 26, and 10 wild-type, *R26Arl13b-Fucci2a*^*+/Tg*^ *and R26Arl13b-Fucci2a*^*Tg/Tg*^ animals, respectively; two-tailed chi-square test; p = 0.6065). *R26Arl13b-Fucci2a*^*Tg/Tg*^ animals were fertile and grossly phenotypically indistinguishable from wild-type littermates (Figures S1E and S1F). Bangs et al. (2015) reported an increase in ciliary length of 37% in mCherry-ARL13B mice without any observed phenotypic consequences. Here, we observed a similar, significant 1.48× increase in ciliary length in *R26Arl13b-Fucci2a*^*Tg/Tg*^ mouse embryonic fibroblasts (MEFs) compared to wild-type MEFs (Figures S2A and S2B) but not in motile multiciliated nasal epithelia (Figures S2C and S2D) or ependymal cells of the adult brain (Figures S2E–S2G). However, a small but significant 1.13× increase in cilia length was noted in adult kidney cortical tubules (Figures S2H–S2J), and a 1.25× increase was observed in liver bile ducts (Figures S2K–S2M). Lengthening of primary cilia in monociliated cells but not of cilia in multiciliated cells may be due to a dilution of ARL13B-Cerulean. Importantly, cilia lengthening as a result of ARL13B overexpression here and in previous models is tolerated *in vivo* and does not interfere with normal development as demonstrated by the grossly normal embryonic and adult phenotypes, including viability, growth, and fertility observed in homozygous *R26Arl13b-Fucci2a*^*Tg/Tg*^ animals (Figures S1F and S2). To our knowledge, our Arl13b-Fucci2a reporter labels all cilia, as evidenced by sensitive detection of diverse cilia types found on neuronal, ependymal, and choroid plexus cells in the adult brain (Figure S3).

## Results

### Cilia Persist after the G1/S Transition in Proliferating NIH 3T3 Cells

**Figure 2:**
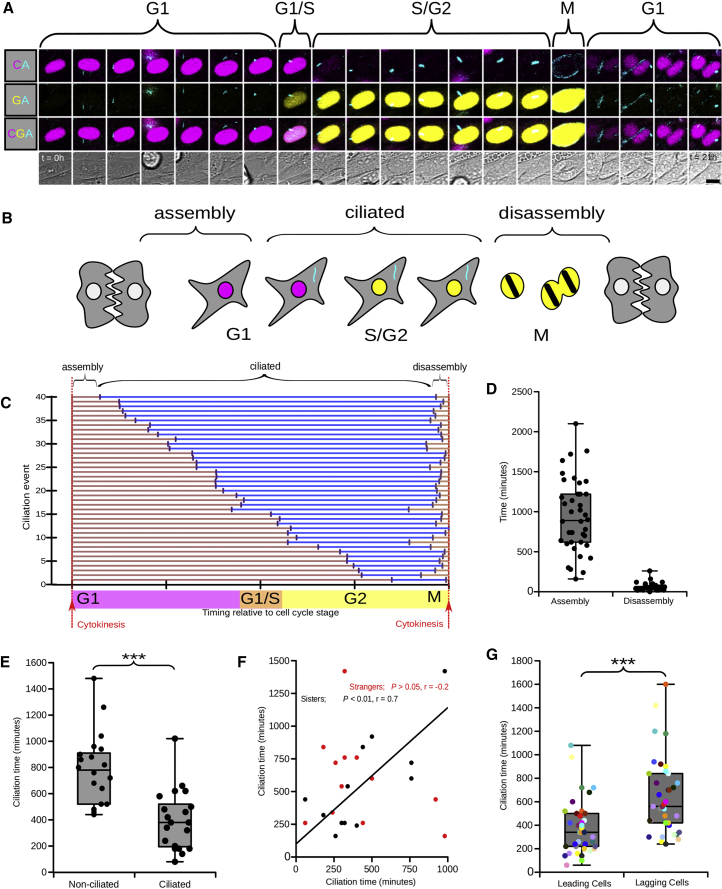
The Dynamics of the Cilia Assembly and Disassembly Cycle in Arl13bCerulean-Fucci2a NIH 3T3 Cells (A) Cell cycle progression of a single Arl13bCerulean-Fucci2a labeled nucleus as it cycles from G1 through S and G2 phases and undergoes mitosis. The sequential peaks of mCherry-hCdt1(30/120) and mVenus-hGem(1/110) are evident as is the presence of a single Arl13b-Cerulean labeled cilium during the G1, S, and G2 phases before being disassembled shortly before mitosis. (B) The dynamics of the cilia cycle in Arl13bCerulean-Fucci2a 3T3 cells were determined in time-lapse experiments. We defined cilia assembly and disassembly times as the time between cytokinesis and the initiation of cilia formation or the completion of cilia resorption (loss of ARL13B-Cerulean localization). (C) Analysis of individual cell ciliation events showing the ciliation state of each cell and its relative position within the G1 and S/G2/M cell cycle phases (n = 40 ciliation events). (D) Analysis of cilia assembly and disassembly times in cycling Arl13bCerulean-Fucci2a 3T3 cells (n = 40) revealed that, while cilia assembly time varied greatly, disassembly happened in a relatively tight window. (E) We compared the behavior of the progeny of ciliated and non-ciliated cells. Cilia assembly is significantly faster after mitosis in the progeny of ciliated mother cells compared to the progeny of non-ciliated mothers (Student’s t test; p < 0.001; n = 20 in each group). (F) Further investigation of the variation in cilia assembly time revealed a positive correlation between the cilia assembly times of the direct daughters of a mitosis (Spearman’s rank; p < 0.01; *r* = 0.7; n = 12 pairs) but not between randomly paired cells (Spearman’s rank; p > 0.05; r = −0.2; n = 12 pairs). (G) Comparing the sisters in individual daughter pairs revealed that one daughter typically lagged behind the other in the time taken to initiate cilia formation; we termed these daughters leading and lagging cells. There is a significant difference in cilia assembly timing between these groups (Student’s t test; p < 0.001; n = 37 daughter pairs). Boxplots indicate minimum, maximum, median, and interquartile range; all values are shown. Scale bar: 10 μm in (A); t = time in hours.

It has been shown in cell synchronization experiments that there are two distinct phases of cilia disassembly (Tucker et al., 1979, Pugacheva et al., 2007). The first occurs 1–2 hr after serum stimulation, with the second occurring after 18–24 hr such that cells can re-enter the cell cycle. However, the precise timing of cilia assembly and disassembly in relation to the cell cycle in actively cycling cells is still unclear and has not been directly investigated by live imaging. In unsynchronized NIH 3T3 cells, we frequently observed mVenus-hGem(1/110) positive cells (in S/G2/M) that harbored ARL13B-Cerulean labeled cilia (Figure 1B). To investigate this further, time-lapse imaging of cultured Arl13bCerulean-Fucci2a 3T3 cells was performed. We observed cilia assembly and disassembly and measured the timing of these events in parallel to Fucci2a cell cycle status and in relation to cytokinesis (Figures 2A–2D). The mean time for completion of cilia disassembly, as defined by complete loss of ARL13B-Cerulean localization, was 65.50 ± 17.07 min prior to cytokinesis (Figure 2D). Often, loss of ARL13B-Cerulean localization occurred immediately before (within the image acquisition time of 20 min per frame) the breakdown of the nuclear envelope (Video S1, right panel). By defining the proportion of each cell cycle phase in which a cell was ciliated, we were able to examine both the onset of ciliation and the point of resorption in detail (Figure 2C). To our surprise, we observed cells initiating cilia formation at any point from early G1 through to mid S/G2/M with a mean (± 95 % CI) of 948.00 ± 140.62 min, while resorption was restricted close to cytokinesis (Figures 2C and 2D).

### The Propensity to Ciliate and the Timing of Ciliation Are Both Heterogeneous and Heritable in NIH 3T3 Cells

One cause of variation in ciliation time was the significantly faster ciliary assembly seen in daughter cells derived from a ciliated mother compared to those derived from non-ciliated mothers (Figure 2E). It is possible the intracellular metabolic environment inherited by daughter cells from a ciliated mother could prime them for early ciliogenesis. Reflecting this, a strong correlation was observed in the time taken for cilia assembly between sisters of a mitosis in the next cilia cycle (Figure 2F; Video S1, right panel). Interestingly, although the timing of ciliary assembly between sisters was well correlated (Figure 2F), when the mean time taken for the first and second sisters to ciliate was compared, a statistically significant difference was observed (Figure 2G). We hypothesize that this disparity reflects the proposed early formation of cilia in the sister that inherits the mother centriole (Anderson and Stearns, 2009, Paridaen et al., 2013). Taken together, these data highlight that there is heterogeneity in the propensity to ciliate in NIH 3T3 cells and that this propensity is in part directly heritable.

### The Nuclear-Ciliary Angle Correlates with Directed Movement in Migrating NIH 3T3 Cells

**Figure 3:**
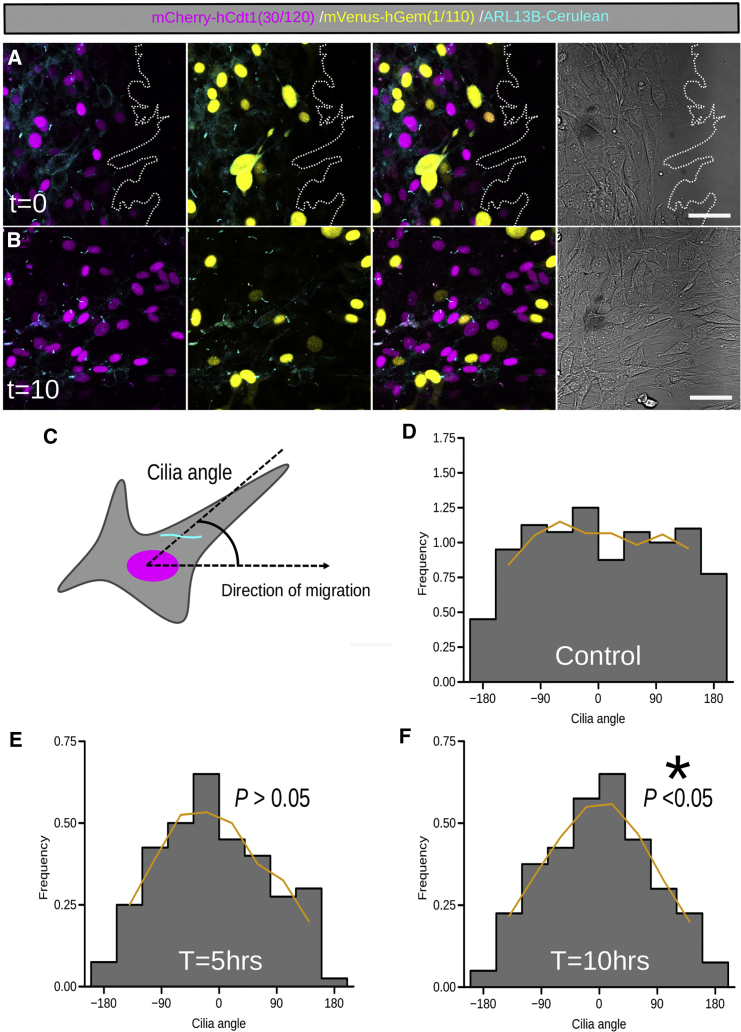
Arl13bCerulean-Fucci2a 3T3 Cells Orientate Their Cilia toward the Leading Edge Concurrent to Migration during Wound Healing (A) Images taken of Arl13bCerulean-Fucci2a 3T3 cells migrating into the cell free space after the removal of a silicon barrier. (B) Images of the region shown in (A) after 10 hr of wound healing. (C) The orientation of the primary cilium was calculated for individual cells by measuring the angle between the center of the cell’s nucleus and the cilium, followed by normalization to the collective angle of migration. (D) A histogram of ciliary angles in a control experiment in which there is no directional movement shows a uniform distribution of ciliary angles (n = 387 cilia). (E) In the migration assay, 5 hr after removal of the silicon barrier, the majority of cells have reoriented their primary cilia so that the distribution of angles coalesces around the direction of migration (n = 133 cilia). (F) After 10 hr, the distribution of cilia angles was shown to differ significantly from the uniform distribution observed in the control experiment (two sample Kolmogorov-Smirnov test; p < 0.05). Scale bars: 50 μm in (A) and (B).

Primary cilia are known to be orientated between the nucleus and the leading edge during cell migration (Christensen et al., 2013, Schneider et al., 2010). It is not known whether this process begins before cell migration or whether it is concomitant with cell movement. To understand how a cell may adopt this orientation, we performed time-lapse imaging of Arl13bCerulean-Fucci2a NIH 3T3 cells in a modified wound-healing assay (see STAR Methods; Figures 3A and 3B; Video S2). The angle between the primary cilium-to-nucleus and the nucleus-to-wound edge was determined 5 and 10 hr after wound induction (Figures 3C–3G) and compared to a control experiment where cells were seeded evenly onto a plate with no stimulus. We observed a homogeneous distribution of ciliary angles in the control group (Figure 3D). In response to the wound healing stimulus, there was a significant reorientation of cilia perpendicular to the leading edge after 10 hr in culture (Figures 3E and 3F).

Video S2. Ciliated Migrating Arl13bCerulean-Fucci2a NIH 3T3 Cells in a Migration Assay, Related to Figure 3

### Scission of Mature Ciliary Tips Is Required to Stabilize Cilia in NIH 3T3 Cells

**Figure 4:**
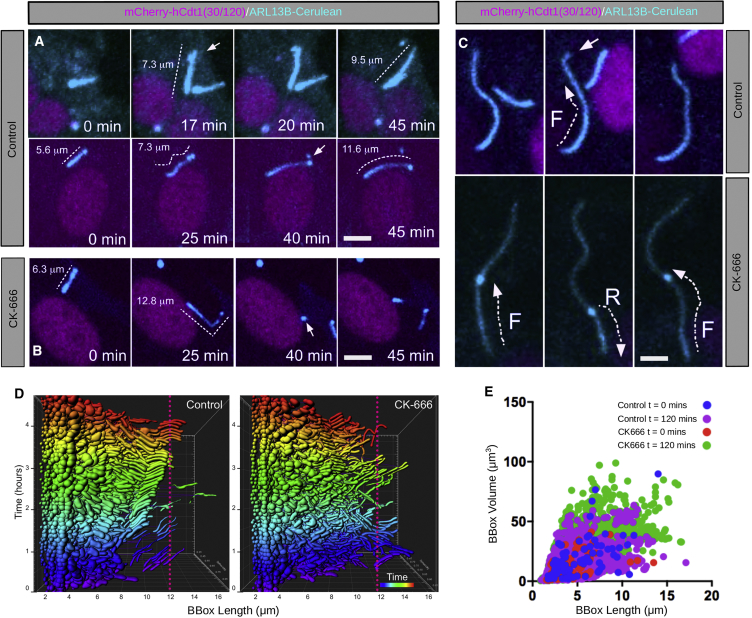
Ciliary Decapitation Occurs during Ciliary Growth in G1 and Is Dependent on F-Actin (A) Live imaging of Arl13bCerulean-Fucci2a NIH 3T3 cells in G1 (mCherry-hCdt1(30/120) positive) showing cilia decapitation (arrows at 17 and 40 min) prior to cilia elongation. (B) The addition of 200-μM F-actin inhibitor (CK-666) destabilized cilia resulting in frequent decapitations (arrow at 40 min) far from the scission point. (C) Cilia swellings moving in the anterograde direction (F) were occasionally observed prior to cilia scission. In the presence of CK-666, swellings were observed moving in both anterograde (F) and retrograde (R) directions. (D) The bounding box (BBox—minimum bounding rectangular cuboid) of each cilium was identified using image processing, and a comparison of the BBox morphology of elongating cilia after serum starvation was performed. Cilia assembling in the presence of CK-666 are less stable and have a deformed morphology compared to controls. (E) Scatterplot correlating the bounding box length and volume of elongating cilia at the start and end of the time-lapse experiment in (D). Comparison of the ratio of volume to length showed a statistically significant difference between the control and CK-666 treated groups at 120 min (2-way ANOVA; p < 0.0001; Tukey’s HSD; p < 0.0001). Scale bars: 5 μm in (A)–(C); BBox = bounding box.

It has recently been shown that the tip of a primary cilium is actively severed in order to clear activated ciliary receptors (Nager et al., 2017) and to initiate ciliary disassembly (Phua et al., 2017). This scission process is mediated through an F-actin-dependent mechanism (Nager et al., 2017, Phua et al., 2017) termed ectocytosis (Wood et al., 2013). In agreement with this proposed mechanism, mutants with compromised actin dynamics have delayed ciliary disassembly (Yeyati et al., 2017). In these “ectocytosis mutants,” ciliogenesis is facilitated, but ciliary stability is reduced, resulting in an abnormally wide variation in ciliary lengths, suggesting that actin-mediated ectocytosis plays a broader and constitutive role in the maintenance of cilia (Nager et al., 2017, Yeyati et al., 2017). To investigate this hypothesis, we observed cilia growth during serum depletion in Arl13bCerulean-Fucci2a NIH 3T3 cells. By focusing on cells labeled with mCherry-hCdt1(30/120) (G1 phase only), we excluded ciliary ectocytosis events that would normally precede resorption during G2 phase prior to mitosis. In the absence of serum, we consistently found ciliary elongation after scission in G1 cilia (Figure 4A; Video S3), confirming that ciliary scissions are not limited to cilia committed to resorb but can also occur under conditions that promote ciliary growth. We tested the dependence of G1-ectocytosis on actin dynamics through inhibition of the ARP2/3 complex activity, previously shown to participate in an F-actin ciliary gate (Yeyati et al., 2017). In control cultures, scission occurred mostly at the ciliary tip (38 cilia; 10/12 excisions occurring at tip) and was in some instances preceded by discernible ciliary swellings. Conversely, in the presence of the ARP2/3 inhibitor CK-666, cilia rapidly elongated, breaking frequently at the base and away from the tip (32 cilia; 4/16 excisions occurring at tip; Figure 4B; Video S4) with bulges that often persisted throughout the time-lapse traveling repeatedly in anterograde or retrograde directions (Figure 4C; Video S5). Morphometric comparisons confirmed that while control cilia present orderly growth and homogeneous morphology, CK-666-treated cilia changed shape rapidly and presented a more disordered morphological profile (Figures 4D and 4E). The results further provide evidence that actin-mediated trimming of the ciliary tip is operational all along the ciliary cycle and contributes to ciliary stability.

Video S3. Scission of Primary Cilia in G1 Arl13bCerulean-Fucci2a NIH 3T3 Cells after Serum Starvation, Related to Figure 4

Video S4. Destabilization of Primary Cilia after Addition of the F-Actin Inhibitor CK-666 in Serum-Starved Arl13bCerulean-Fucci2a NIH 3T3 Cells, Related to Figure 4

Video S5. Anterograde and Retrograde Movement of Cilia Bulges in Arl13bCerulean-Fucci2a NIH 3T3 Cells Treated with CK-666, Related to Figure 4

### Arl13bCerulean-Fucci2a Identifies the Node as a Cluster of Ciliated Cells in G1/G0

Left-right asymmetry is established in the early mouse embryo by the action of motile cilia in a specialized embryonic organizer structure known as the node located at the anterior end of the primitive streak (Lee and Anderson, 2008). The beating of these nodal cilia is required to generate nodal flow to break the left-right symmetry of the developing embryo (Nonaka et al., 1998, McGrath et al., 2003, Hirokawa et al., 2006, Becker-Heck et al., 2011, Shinohara et al., 2012, Yoshiba et al., 2012). This flow may be detected directly through a mechanosensory mechanism (Shinohara et al., 2012) or may in turn generate a gradient of chemokines or chemokine containing “nodal vesicular particles” (Tanaka et al., 2005). Sensing of this gradient or flow may be achieved by specialized nonmotile cilia at the border or “crown” of the node initiating asymmetric calcium signaling at its left margin (McGrath et al., 2003).

**Figure 5:**
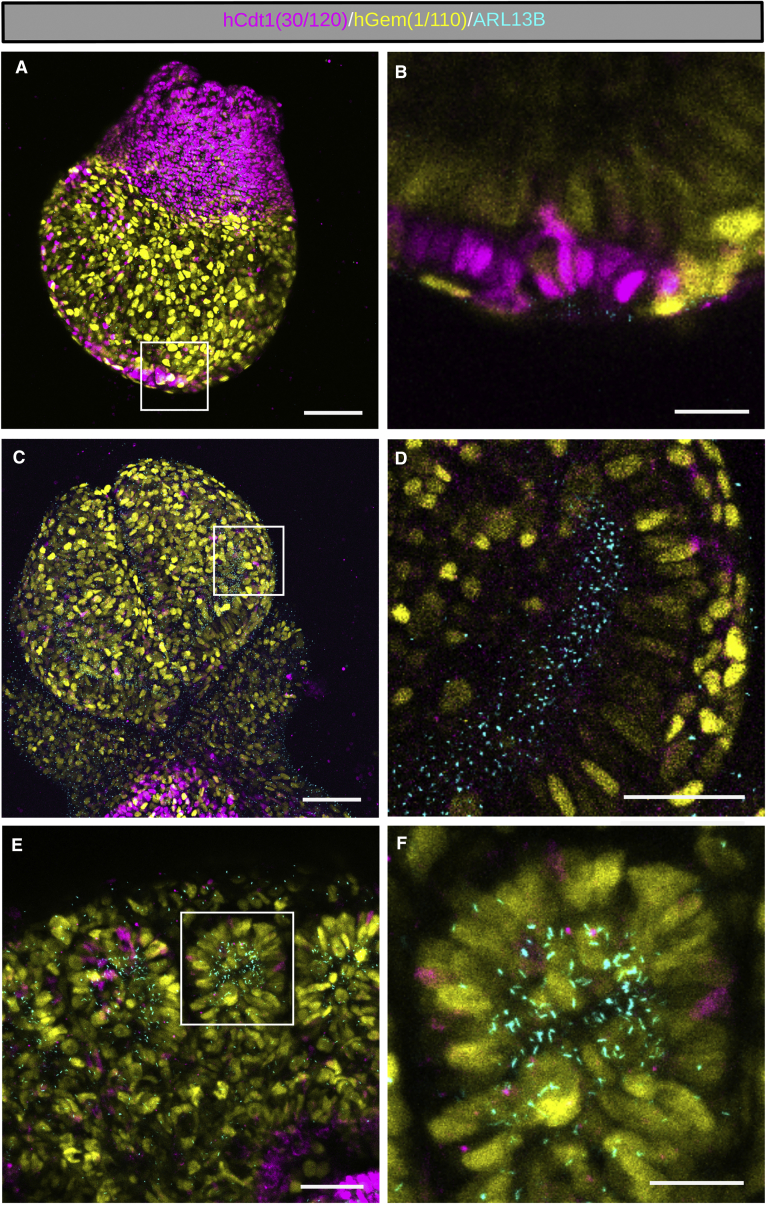
Visualization of Primary Cilia and Cell Cycle Status in *R26Arl13b-Fucci2aR*^*+/Tg*^*; CAG-Cre*^*+/Tg*^ Embryos Ubiquitous expression of the Arl13bCerulean-Fucci2a transgene was achieved by crossing *R26Arl13b-Fucci2aR* mice with ubiquitous *CAG-Cre* mice, followed by whole-mount confocal imaging of E7.5 and E8.5 embryos. (A) A representative Z-projection of a neural plate stage E7.5 *R26Arl13b-Fucci2aR*^*+/Tg*^*; CAG-Cre*^*+/Tg*^ embryo. Cells of the proximal extraembryonic ectoderm are predominantly labeled with mCherry-hCdt1(30/120) and are starkly less proliferative compared to the distal embryonic visceral endoderm and epiblast lineages that contain a large proportion of cells in S/G2/M phases of the cell cycle labeled with mVenus-hGem(1/110). In all cases (n = 4 *R26Arl13b-Fucci2a*^*+/Tg*^ E7.5 embryos from 3 litters), the node can be identified as a cluster of mCherry-hCdt1(30/120) positive cells at the distal pole of the embryo (boxed area). (B) A single plane of boxed area in (A) showing the node as a minor population of cells in G1/G0. Cells of the node are orientated perpendicular to the embryonic surface with mCerulean-ARL13B positive cilia pointing into a concave depression at the distal tip of the embryo (also see Figure S2). (C) A representative Z-projection of a 5S stage E8.5 *R26Arl13b-Fucci2a*^*+/Tg*^*; CAG-Cre*^*+/Tg*^ prosencephalon (n = 8 *R26Arl13b-Fucci2a*^*+/Tg*^ E8.5 embryos from 2 litters). The majority of cells on the surface and within the future forebrain are mVenus-hGem(1/110) positive, many of which are ciliated. (D) A single plane of the boxed area in (C) showing a lateral ventricle. A high density of cilia are located in the lumen of the ventricle surrounded by mostly mVenus-hGem(1/110) positive neuroepithelial cells orientated perpendicular to the lumen (also see Figure S3). (E) A representative Z-projection of the rhombencephalic region of an E8.5 *R26Arl13b-Fucci2aR*^*+/Tg*^*; CAG-Cre*^*+/Tg*^ embryo (n = 8 *R26Arl13b-Fucci2a*^*+/Tg*^ E8.5 embryos from 2 litters). Emerging somites are clearly distinguishable as clusters of cycling cells labeled with mVenus-hGem(1/110) and mCherry-hCdt1(30/120). A high density of cilia are seen within each somite associated with cells in both G1 and S/G2/M phases of the cell cycle. Primary cilia are also identifiable on cycling cells outside of each somite. (F) Increased magnification of a single somite from boxed region in (E) (also see Figure S4). Scale bars: 100 μm in (A), (C), and (E). Scale bars: 50 μm in (B) and (D), and 25 μm in (F).

We performed whole-embryo live imaging of embryonic day (E) 7.5 *R26Arl13b-Fucci2aR*^*+/Tg*^*; CAG-Cre*^*+/Tg*^ embryos (n = 4; Downs and Davies stages: late streak [LS]-early headfold [EHF]) and were able to distinguish cell cycle stage-specific abundance of the Fucci2a probes and localization of ARL13B-Cerulean to primary cilia. As expected, our results revealed a clear demarcation in cell cycle status between the extraembryonic visceral endoderm and the embryonic visceral endoderm and epiblast (distal) lineages (Figures 5A and S4A–S4D). The majority of cells within the embryonic lineages were proliferating and therefore mVenus-hGem(1/110) positive. However, as previously reported (Komatsu et al., 2011), the cells of the node were clearly identifiable as a cluster of mCherry-hCdt1(30/120) positive cells (in G1/G0) located at the tip of the embryo (Figures 5A, 5B, and S4A–S4F). Here, nodal cilia are orientated in what will become a 10-μm deep concaved compartment, the nodal pit, discernible by ∼E7.75 (Lee and Anderson, 2008). By turning the embryo and imaging directly into the node, approximately 180 nodal cilia labeled with ARL13B-Cerulean could be identified, consistent with previously reported numbers (200–300) (Shinohara et al., 2012) (Figures S3G–S3I). We were unable to discriminate between pit and crown cilia at this stage as they do not become spatially distinct until slightly later. *R26Arl13b-Fucci2aR*^*Tg/+*^ heterozygous mice therefore express the Arl13bCerulean-Fucci2a biosensor at levels high enough to enable high-resolution live confocal imaging, allowing discrimination of individual cells by cell cycle stage and the identification of cilia across lineages.

### Arl13bCerulean-Fucci2a Reports on Primary Cilia Dynamics and Cell Cycle Stage during Organogenesis

To analyze cilia and the cell cycle during organogenesis, we performed live imaging of whole-mount E8.5 embryos (n = 8, Downs and Davies stages: 0 somites [S]-8S stage) revealing the majority of cells to be ciliated, regardless of cell cycle stage (Figures 5C and S5A–S5C). Very few cells within the prosencephalon were mCherry-hCdt1(30/120) positive at this stage, suggesting a high rate of proliferation (Figure S5A). Confocal sectioning revealed the lateral ventricles surrounded by an epithelium of highly proliferative cells, as shown by the high proportion of cells labeled with mVenus-hGem(1/110), orientated perpendicular to the lumen (Figures 5D and S5D–S5I; Video S6). The lumen of the lateral ventricles contained a high density of primary cilia projecting from the surrounding neuroprogenitor cells (Figures 5D and S5G–S5I).

Video S6. Optical Confocal Sectioning of an E8.5 *R26Arl13b-Fucci2aR*^*Tg/+*^; *CAG-Cre*^*Tg/+*^ Mouse Forebrain, Related to Figure 5

Within the E8.5 rhombencephalic region, the first somites were identifiable as morphologically distinct segmented groups of cells, predominantly in S/G2/M phases of the cell cycle (Figures 5E, 5F, and S6). Cilia were clearly identifiable on cells surrounding the somites in G1 and S/G2/M stages of the cell cycle (Figures 5E, 5F, and S6A–S6C). Within each somite, a high density of primary cilia were located projecting into the central core (Figures 5F and S6D–S6F). The presence of primary cilia within both emerging and mature somites coincides with the induction of sclerotome development by SHH gradients (Fan and Tessier-Lavigne, 1994). *R26Arl13b-Fucci2aR* is the first model allowing simultaneous imaging of cilia and cell cycle progression during development. Our *in vivo* observations suggest that cilia persist through the G1/S transition and into S/G2/M phase during development in agreement with our observations in actively cycling NIH 3T3 cells in culture (Figure S7).

### Live Imaging of Primary Arl13bCerulean-Fucci2a Labeled Cells Captures Cilia Dynamics during Differentiation and Regeneration

**Figure 6:**
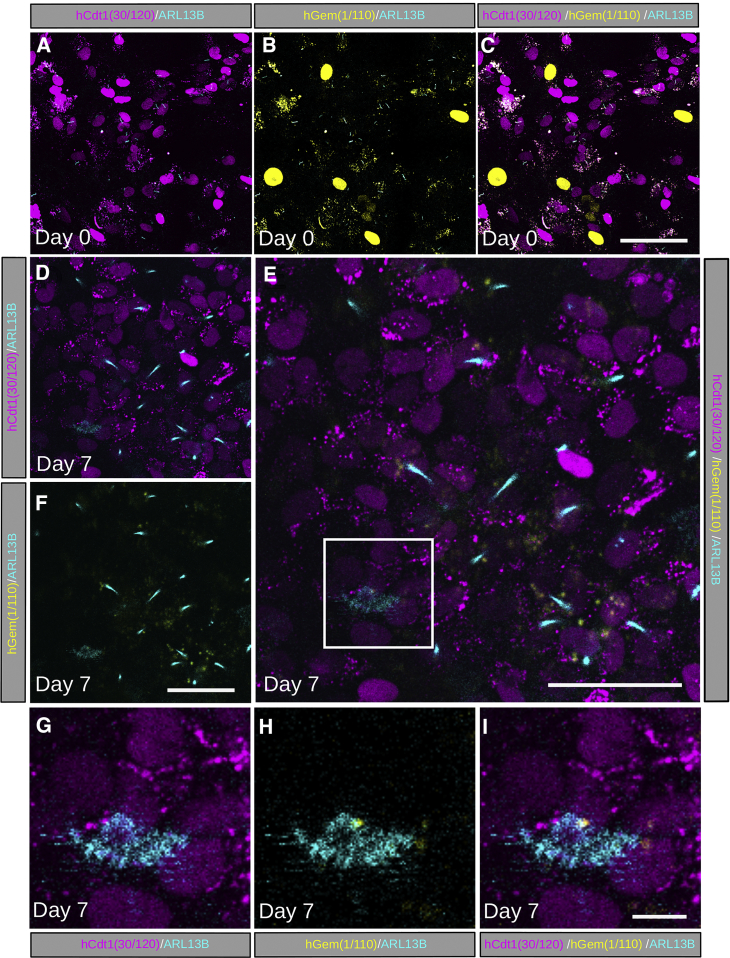
Primary Ependymal Cultures Exit the Cell Cycle and Form Multiple Motile Cilia during Differentiation Primary ependymal cultures were prepared from the ventricular zone of E18.5 *R26Arl13b-Fucci2aR^+/Tg^; CAG-Cre^+ve^* embryos. Primary cell cultures were grown to confluency before being serum starved (Day 0) to induce differentiation followed by live confocal imaging. (A) At Day 0, ciliated cells in G1 phase of the cell cycle labelled with mCherry-hCdt1 (30/120). (B) Area in (A) showing ciliated cells in S/G2/M phase of the cell cycle labelled with mVenus-hGem (1/110). (C) Merge of area imaged in (A,B) showing primary cilia are present on a subset of cells in both G1 and S/G2/M phase. (D) Upon reaching confluency and following 7 days of serum starvation, all cells had dropped out of the cell cycle and were mCherry-hCdt1 (30/120) positive. (E) Merged image (D, F) showing emergence of Cherry-hCdt1 (30/120) positive multiciliated cells. (F) After 7 days serum starvation, no cells were positive for mVenus-hGem (1/110). (G) Magnification of box in (E) showing mCherry-hCdt1(30/120) labelled cells with motile multicilia labelled with ARL13B-Cerulean (also see Video S8). (H) Ependymal cells had exited the cell cycle as shown by lack of mVenus-hGem (1/110) expression. (I) Merged image (G, H). Scale bars: 50 μm in (C), (F), and (E), and 10 μm in (I).

To use our *R26Arl13b-Fucci2aR* mice as a tool to visualize the interplay between cilia types and cell cycle status during differentiation, we turned our attention to the emergence of motile cilia on differentiated cells. Multiciliated primary ependymal cultures were derived from the ventricular zone of E18.5 *R26Arl13b-Fucci2aR^+/Tg^; CAG-Cre^+/Tg^* embryos. In early expanding cultures, ependymal cells were proliferative as demonstrated by the presence of cells in S/G2/M phases of the cell cycle, with ARL13B-Cerulean positive primary cilia identifiable on the majority of cycling cells (Figures 6A–6C). Upon reaching confluency and serum starvation, all cells entered G1/G0 labeled with mCherry-hCdt1(30/120). Upon differentiation (7 days after serum starvation), the absence of cycling mVenus-hGem(1/110) ependymal cells and the emergence of cells exhibiting multiple motile cilia (Figures 6D–6I) were observed. High-speed confocal imaging confirmed the motility of these cilia, which moved in a periodic unidirectional whip-like motion required to generate cerebrospinal flow (Video S7).

Video S7. Motile Cilia Beating on Differentiated Primary Mouse Ependymal Cultures Derived from an *R26Arl13b-Fucci2aR*^*Tg/+*^; *CAG-Cre*^*Tg/+*^ Mouse, Related to Figure 5

Cells of the adult hepatic ductal tree are largely quiescent with long monocilia (Figure S2K). These can be isolated to grow organoids, which are genetically stable and can be passaged indefinitely, making them an attractive model for liver disease modeling. We derived hepatic ductal organoids and performed time-lapse imaging. In agreement with the data obtained from Arl13bCerulean-Fucci2a NIH 3T3 cells, we observed many mVenus-hGem(1/110) positive cells simultaneously harboring ARL13BCerulean positive cilia with rapid loss of cilia prior to mitosis and asymmetric rates of ciliation between daughters (Video S8).

Video S8. ***R26Arl13b-Fucci2aR^Tg/Tg^* Adult Biliary Organoids Reveal Cilia Dynamics and Cell Cycle Progression**, Related to Figure 6 Adult bile duct organoids isolated from preps used for Figure S2K (n = 3 animals, 4 months), were expanded and were cultured in 50% growth/50% basal media for 16 hr for imaging, one stack every 20 min. Many mVenus-hGem(1/110)-cells are observed with ARL13B+ cilia. Zoomed-in regions of interest show (1) a rapid loss of cilia just prior to mitosis and (2) asymmetric rates of ciliation between daughters in organoids.

### Endoderm-Restricted Expression of Arl13bCerulean-Fucci2a Reveals the Luminal Protrusion of Primary Cilia during Lung Branching Morphogenesis

**Figure 7:**
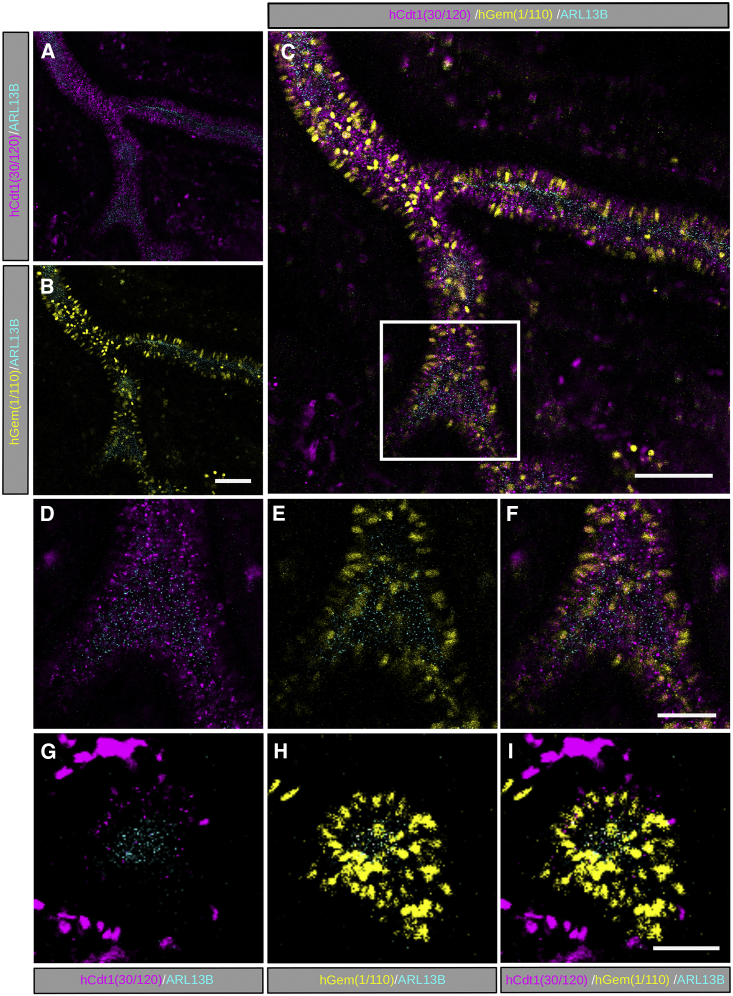
Primary Cilia Line the Luminal Surface of the Airway Epithelium during Lung Development To confirm the ability to induce tissue-specific expression of Arl13bCerulean-Fucci2a, *R26Arl13b-Fucci2aR* mice were crossed with endoderm-specific Cre-recombinase expressing line *Sox17-2A-iCre*. Embryonic lungs were dissected from E11.5 embryos and imaged in ex vivo organotypic culture (n = 9 *R26Arl13b-Fucci2a^+/Tg^* E11.5 embryos from 2 litters). Arl13bCerulean-Fucci2a expression was restricted to the lung epithelium and a subset of migratory mesenchymal cells. (A) In the proximal non-branching regions of the epithelium, cells were predominantly in G1 labelled with mCherry-hCdt1(30/120). ARL13B-Cerulean labelled cilia were visible along the entire length of the airway epithelium. (B) A subset of proximal epithelial cells resided in S/G2/M - labelled with mVenus-hGem(1/110). (C) Merged image (A,B). (D) Magnification of the region in (C) showing mCherry-hCdt1(30/120) labelled epithelial cells with primary cilia oriented towards the lumen of the branching tubule. (E) Magnification of the box in (C) showing mVenus-hGem(1/110) labelled epithelial cells with primary cilia oriented towards the lumen of the branching tubule. (F) Merged region of interest (D,E). (G) At the distal tips of the actively branching epithelium, mCherry-hCdt1(30/120) labelled epithelial cells are largely absent but ARL13B-Cerulean labelled cilia are present projecting into the lumen. (H) A highly proliferative area is evident at the branching tip where mVenus-hGem(1/110) labelled epithelial cells predominate and harbour ARL13B-Cerulean labelled cilia that project into the lumen. (I) Merged image of region of interest (G,H). Scale bars: 50 μm in (B) and (C), and 10 μm in (F).

To demonstrate tissue-specific expression of Arl13bCerulean-Fucci2a in *R26Arl13b-Fucci2aR* mice, we crossed the reporter with endoderm-specific *Sox17-2A-iCre* expressing mice (MGI: 4418897). *Sox17-2A-iCre* mice were previously shown to label all endoderm-derived tissues including the developing lung epithelium as well as the hematopoietic and vascular lineages (Engert et al., 2009). Live imaging of dissected E11.5 lungs after 12 hr of culture revealed restricted lung epithelial expression of Arl13bCerulean-Fucci2a as well as expression in a sub-population of migratory cells (Figures 7A–7C), most likely of the hematopoietic, vasculature, and smooth muscle lineages also derived from SOX17-expressing endoderm (Engert et al., 2009). Cells of the proximal lung epithelium were predominately labeled with mCherry-hCdt1(30/120), consistent with a population exiting the cell cycle and entering G0 (Figure 7A). In contrast, a high level of proliferation indicated by the high proportion of mVenus-hGem(1/110) positive cells was seen in actively branching apical tips of the epithelium (Figures 7G–7I). This is in agreement with previous observations in cultured lungs from *R26Fucci2aR*^*+/Tg*^*; CAG-Cre*^*+/Tg*^ embryos (Mort et al., 2014). Motile cilia in the mouse lung form from E14.5 (Rawlins et al., 2007); however, primary cilia have been identified as early as E12.5 (Jain et al., 2010). Using live imaging of E11.5 lung organotypic cultures, we were able to visualize primary cilia along the entire length of the lung epithelium within the actively branching regions at distal branch tips and within the more proximal airway epithelium where proliferation has ceased. Cilia were observed on the apical surface of the proximal and distal cells, orientated so that they projected into the adjacent lumen (Figures 7D–7I).

## Discussion

Here, we describe the design and validation of an Arl13bCerulean-Fucci2a tricistronic reporter construct encoding optimally spectrally separated biosensors that label both primary and motile cilia with ARL13B-Cerulean and report cell cycle progression through the Fucci2a cell cycle probes (Abe et al., 2013, Mort et al., 2014).

### Unearthing Unexplored Ciliary Parameters

In previous reports, the preference of cells to orientate their primary cilia toward the leading edge during migration and in response to chemoattractant cues was observed, but the timing was not determined (Schneider et al., 2010, Christensen et al., 2013, McGowan and McCoy, 2013). Our results suggest that this reorientation is in part due to the cytoskeletal changes required for directed migration and does not require the presence of a chemoattractant. As well as investigating cilia orientation, Arl13bCerulean-Fucci2a offers the means to easily separate other ciliary events, such as scission, according to cell cycle phase. We propose that ciliary scission does not just precede ciliary resorption but is instead required throughout the ciliary cycle to maintain ciliary length within its normal range. Scission may coordinate the growth rate of the ciliary membrane with that of the axoneme, and the uncoupling of these events may destabilize cilia.

### Persistence of Cilia after the G1/S Transition Is a General Property in Cells and Mice

Previous studies have used serum starvation to synchronize NIH 3T3 or retinal pigment epithelial (RPE1) cells in G1 followed by serum addition to drive cilia disassembly. These studies reported cilia reassembly in G1 and two rounds of cilia disassembly, 1–2 hr and 18–24 hr after the addition of serum. The second round of disassembly occurred around the time of DNA replication (Tucker et al., 1979, Pugacheva et al., 2007, Spalluto et al., 2013). We show here that cilia generally assemble during G1 and that the completion of cilia disassembly is close to cytokinesis, often within 20 min of the breaking of the nuclear envelope at mitosis. This is consistent with previous reports in rat kangaroo kidney epithelial (PtK1) cells showing disassembly in early M-phase (Rieder et al., 1979). The strength of our model is that we can resolve individual cells in physiologically relevant unsynchronized cell populations rather than by using artificial synchronization protocols.

A popular school of thought is that the primary cilium is resorbed or shed at the G1-S transition such that centriole duplication can then proceed (Hinchcliffe and Sluder, 2001, Nigg and Stearns, 2011). However, our data suggest that the presence of a primary cilium and the duplication of the centrioles are not mutually exclusive in NIH 3T3 cells but rather that the mother centriole is acting as the basal body of the cilia while centriole duplication is occurring. Primary cilia were also identified on cells in G1 and S/G2/M stages in all examined tissues in the developing mouse (Figure S7), suggesting that this is a common mechanism. Persistence of cilia into late S/G2/M-phase may result in the sequestering of one set of centrioles at the base of the cilia prior to mitotic spindle formation, influencing the future plane of cell division. Interestingly, kidney-specific deletion of *Ift20* and *Kif3a* misorientates spindle assembly concurrent with cilia loss resulting in epithelial cyst formation and suggesting a potential role of cilia in the positioning of the mitotic spindle (Jonassen et al., 2008, Patel et al., 2008). Many cilia- and centriole-associated proteins have cilia-independent functions essential for correct spindle formation and cytokinesis (Vertii et al., 2015). Therefore, careful dissection of cilia-dependent and independent functions is required to determine the importance of primary cilia in spindle orientation.

### Tracking Ciliary Assembly and Disassembly and the Heritability of a Primed Ciliary State

Our data support previous reports that the mother centriole can prime a cell to ciliate (Anderson and Stearns, 2009). This phenomenon may determine fate choice decisions in embryonic neocortical stem cells during neurogenesis (Paridaen et al., 2013). The inheritance of the mother centriole in neocortical progenitors, identified by the association with remnants of the ARL13B^+^ cilia membrane, enabled one daughter to reassemble its primary cilia faster and asymmetrically retain its position in the stem cell niche, while the second daughter was destined for differentiation. The ARL13B-Cerulean model now provides a tool to assess whether this might be a common mechanism in asymmetric cell fate decisions *in vitro* and *in vivo*.

### A Mouse Reporter for Combined Live Imaging of Ciliogenesis and the Cell Cycle

In agreement with previous studies, we could clearly distinguish the embryonic node at E7.5 as a collection of mCherry-hCdt1(30/120) positive cells harboring ARL13B-Cerulean labeled cilia at the anterior tip of embryo (Komatsu et al., 2011). In the E8.5 prosencephalon, neuroepithelial cells surrounding the lateral ventricles projected their primary cilia into the luminal space reminiscent of the orientation of primary cilia during neurogenesis (Paridaen et al., 2013). This developmental stage precedes neurogenesis, which commences around E11; here, the neuroepithelium surrounding the ventricles is constructed of a single layer of neural stem cells organized into a pseudostratified neuroepithelium (Götz and Huttner, 2005). It has been shown that during neurogenesis the cerebrospinal fluid (CSF) provides proliferative and survival support to neural progenitors by IGF2 signaling with binding enriched along primary cilia (Lehtinen et al., 2011). It is possible the anchoring of cells to the ventricular lumen via a ciliated membrane at E8.5, prior to neurogenesis, may also be required to detect signals in the CSF necessary to maintain their multipotent potential and promote proliferation.

SHH released from the notochord and floor plate has previously been shown to be necessary for somitogenesis by promoting proliferation and expression of the sclerotomal markers PAX1, PAX9, and SOX9 via the activity of the SHH signaling GLI transcription factors (Buttitta et al., 2003, Chiang et al., 1996, Fan and Tessier-Lavigne, 1994, Fan et al., 1995, Murtaugh et al., 1999, Zeng et al., 2002). Our identification of a high density of primary cilia within the developing somites suggests these cells are competent to receive and interpret the SHH signals required for their growth and differentiation.

### Potential Roles for Ciliation in Lung Branching Morphogenesis

Primary cilia have been identified on the luminal surface of E12.5 proximal lung epithelial cells prior to the appearance of multiple motile cilia from E15.5 on post-mitotic airway epithelial cells (Jain et al., 2010). Here, we report the presence of primary cilia along the entire length of the luminal aspect of the lung epithelium during branching morphogenesis in cultured E11.5 lungs, including the most distal, actively branching, and highly proliferative regions. The role of primary cilia during branching morphogenesis in the lung has not been addressed. However, in the mammary gland, disruption of ciliogenesis in double *Kif3a*/*Ift20* mutants results in severe branching defects in addition to increased canonical WNT and decreased SHH signaling (McDermott et al., 2010). An intriguing question is whether primary cilia have a similarly important role during lung epithelial branching. In the lung, SHH expression in the distal epithelium attenuates FGF10 released from the surrounding mesenchyme and is required for branch formation (Bellusci et al., 1997, Warburton et al., 2005). It is possible that sequestration of primary cilia to the luminal surface prevents autocrine cilia-dependent signaling. Another intriguing role for primary cilia during lung branching morphogenesis could be in the establishment of epithelial planar cell polarity (PCP). It has been reported that the intraflagellar transport protein *Ift88* (essential for primary cilia assembly) is required for establishing PCP for convergent extension during mouse cochlear development (Jones et al., 2008), suggesting a potential link between primary cilia and PCP, a process important for epithelial fold formation. In the lung, mice harboring mutations in the PCP genes *Celsr1* or *Vangl2* exhibit reduced and misshapen branching (Yates et al., 2010). It would be interesting to evaluate the potential role of lung epithelial primary cilia during branching morphogenesis by conditional deletion of cilia in the lung epithelium.

### Limitations

*R26Arl13b-Fucci2aR* mice are the best available model for tracking the cell and cilia cycles with live imaging; however, there are several limitations of the technology. First, because only the nuclei and the ciliary membrane are labeled, it can be hard, especially in densely labeled static images, to correlate each cilium to a cell body. This could be circumvented in live tissues by applying an appropriately chosen vital dye that preferentially labels the plasma membrane such as wheat germ agglutinin (Fu et al., 2013) or by inducing expression in only a subset of cells using a CreERT2 expressing mouse line and titrating the tamoxifen dose. Second, although in all cells and tissues examined, ARL13B-Cerulean appeared a ubiquitous marker of primary and motile cilia, we cannot rule out the existence of ARL13B-negative cilia where ARL13B-Cerulean does not localize correctly. Third, although there appears to be no gross phenotypic effect on the development, health, or reproduction of homozygous Arl13b-Fucci2a-expressing animals, we cannot rule out subtle changes in cilia-based signaling due to ARL13B overexpression. To circumvent this, it may be possible to directly genome edit the *R26Arl13b-Fucci2aR* reporter mice to carry either of two mutations in the GTPase domain of ARL13B-Cerulean, T35N and R78Q, which localize to cilia without affecting length when overexpressed (Lu et al., 2015).

### Conclusion

We show here that persistence of cilia after the G1/S transition is a general property in proliferative cell populations. The *R26Arl13b-Fucci2aR* reporter mouse uniquely allows inducible, cell type-specific expression of the Arl13bCerulean-Fucci2a biosensor, labeling both primary and motile cilia. The tricistronic nature of the *R26Arl13b-Fucci2aR* reporter mouse will allow researchers to reduce, refine, and replace animals in their future research strategies by simplifying their genetic crosses and keeping fewer animals on the shelf. Tissue-restricted expression should prove particularly useful in developmental studies where lineage tracing is required and where it may be important to distinguish between cycling and non-cycling cells within a ciliated population.

## STAR★Methods

### Key Resources Table

**Table:** 

REAGENT or RESOURCE	SOURCE	IDENTIFIER
**Antibodies**		

Adenylyl Cyclase III (N-14)	Santa Cruz	# sc-32113; RRID:AB_2223118
Arl13b	Proteintech	#17711-1-AP; RRID:AB_2060867
Acetylated α-Tubulin (clone 6-11 B-1)	Sigma	#T6793; RRID:AB_477585
Cytokeratin 19	DSHB	#TROMA-III; RRID:AB_2133570
DNAI2 (1C8)	Sigma	#WH0064446M1; RRID:AB_1841385
GFP	GeneTex	cat# GTX113617; RRID:AB_1950371

**Chemicals, Peptides, and Recombinant Proteins**		

N2 supplement	Gibco	Cat# 17502048
B-27 supplement	Gibco	Cat# 17504044
PD0325901	Stemgent	Cat# 04-0006-02
CHIR99021	Stemgent	Cat# 04-0004-02
CK-666	Sigma	Cat# SML0006
Chir99021 (GSK-3β inhibitor)	Miltenyl	Cat# 130-103-926
Y-27632 (Rock inhibitor)	Tocris	Cat# 1254
A83-01 (TGFβ inhibitor)	Sigma	Cat# SML0788
FGF10	Peprotech	Cat# AF-100-26

**Critical Commercial Assays**		

Flp-In Complete System	Life Technologies	Cat# K601001
Antibleaching live cell visualization medium DMEM^gfp^-2 kit	Evrogen	Cat# MCK02

**Experimental Models: Cell Lines**		

Flp-In 3T3	Life Technoogies	Cat# R76107
Arl13bCerulean-Fucci2a 3T3	This paper	Riken BRC RCB5029
E14 *R26Arl13b-Fucci2aR* ES	This paper	Riken BRC RCB5034
E14 *R26Arl13b-Fucci2a* ES	This paper	Riken BRC RCB5034
Primary ependymal *R26Arl13b-Fucci2a*	This paper	N/A
Primary embryonic fibroblasts *R26Arl13b-Fucci2a*	This paper	N/A
Primary adult bile duct organoids *R26Arl13b-Fucci2a*	This paper	N/A

**Experimental Models: Organisms/Strains**		

Mouse: *R26Arl13b-Fucci2aR (Cre inducible line)* (*Gt(ROSA)26Sor*^*tm1.1(CAG-Cerulean/Arl13b,-Venus/GMNN,-Cherry/CDT1)Rmort*^); maintained on a C57BL6/J background	This paper; EMMA and Riken BRC	MGI:6193734; RBRC10444
Mouse: *R26Arl13b-Fucci2a (Ubiquitous)* (*Gt(ROSA)26Sor*^*tm1(CAG-Cerulean/Arl13b,-Venus/GMNN,-Cherry/CDT1)Rmort*^); maintained on a C57BL6/J background	This paper; EMMA and Riken BRC	MGI:6193732; RBRC10445
Mouse: *R26-Fucci2a (Ubiquitous)* (*Gt(ROSA)26Sor*^*tm1.1(CAG-Venus/GMNN,-Cherry/CDT1)Jkn*^); maintained on a C57BL6/J background	Mort et al. (2014); EMMA and Riken BRC	MGI:6193738; RBRC06511
Mouse: *CAG:Cre*	D.A. Kleinjan, unpublished data	N/A
Mouse: *Sox17-2A-iCre*	Engert et al. (2009)	MGI:4418897

**Oligonucleotides**		

Primers used for cloning see Table S1	This paper	N/A
Primers used for screening ES cells see Table S1	This paper	N/A
Primers used for genotyping mice see Table S1	This paper	N/A

**Recombinant DNA**		

pCDNA5/Frt	Life Technologies	Cat# V601020
POG44	Life Technologies	Cat# V600520
pCAG-H2BCerulean-Fucci2a	R.L.M., unpublished data	N/A
pCAG-Arl13bCerulean-p2a-Fucci2a	This paper; Riken BRC	RDB16059
pCDNA5-CAG-Arl13bCerulean-Fucci2a	This paper; Riken BRC	RDB16057
pCAG-Fucci2a	Mort et al. (2014); Riken BRC	RDB13080
pRosa26-CAG-floxNeo-Fucci2a	Mort et al. (2014); Riken BRC	RDB13081
pRosa26-CAG-floxNeo-Arl13bCerulean-Fucci2a	This paper; Riken BRC	RDB16058
PGK-Cre	Addgene	#11543
pmKate2-N	Evrogen	Cat# FP182

**Software and Algorithms**		

Fiji	Schindelin et al. (2012)	PMID: 22743772
Imaris V9.1	Bitplane	N/A
Nis-Elements AR V4.6	Nikon Instruments	N/A
R-project	R-project	N/A

### Contact for Reagent and Resource Sharing

Further information and requests for resources and reagents should be directed to and will be fulfilled by the Lead Contact, Richard L. Mort (r.mort@lancaster.ac.uk).

### Experimental Model and Subject Details

#### Mouse Strains

All animal work was approved by a University of Edinburgh internal ethics committee and was performed in accordance with institutional guidelines under license by the UK Home Office (PPL 60/4424 and PPL 60/3785). Mice were maintained in the animal facilities of the University of Edinburgh. The *Sox17-2A-iCre* mouse line has been previously described (MGI: 4418897) (Engert et al., 2009). Ubiquitous *CAG-Cre* mice used for germline activation of transgenes were generated by Dr D. A. Kleinjan (The University of Edinburgh) and were previously used in Mort et al. (2014). *R26Arl13b-Fucci2aR* (MGI:6193732) and *R26Arl13b-Fucci2a* (MGI:6193734) mice were maintained on a C57BL/6J background, sex-matched and age-matched C57BL/6J stock animals were used as controls in adult analyses for changes in cilia length. Mice were genotyped using PCR as detailed below, they were housed in a barrier facility with 12 hour light and dark cycles.

#### Cell Lines

Mouse Flp-In NIH 3T3 cells (male - Life Technologies) were cultured in Dulbecco’s modified Eagle’s medium (DMEM) containing; 10% fetal calf serum (FCS), 1% Penicillin/Streptomycin, 25 mM D-Glucose, 4 mM L-glutamine, 1 mM sodium pyruvate and 100 μg/ml Zeocin (Gibco). Mouse E14Tg2A embryonic stem cells (from mouse strain 129/Ola) were cultured in Glasgow’s modified Eagle’s medium (GMEM BHK-21) containing 10% FBS, 1% Sodium Pyruvate, 1% MEM non-essential amino acids, 2mM Glutamine, 0.1mM 2-Mercaptoethanol and 106 U/L LIF (prepared in house). Mouse embryonic fibroblasts were isolated from E12.5 embryos and maintained in Opti-mem (GIBCO), 10% v/v FCS, 1% v/v Penicillin/Streptomycin, 0.1mM 2-Mercaptoethanol (Sigma). All cell lines were maintained in a humidified incubator at 37°C supplied with 5% CO_2_ in air.

### Method Details

#### Construct Design

The Fucci2 cell cycle probe pair consists of a fusion of mCherry with a truncated human CDT1 containing amino acids 30-120 and a fusion of mVenus and the 110 amino acid N-terminus of the human Geminin protein (Abe et al., 2013). The bicistronic Fucci2a construct consists of the Fucci2 probes fused with a *Thosea asigna* virus 2A peptide (T2A) so that mCherry-hCdt1(30/120) is 5’ of the T2A sequence and mVenus-hGem(1/110) is 3’, therefore ensuring proper nuclear localisation of both probes (Mort et al., 2014).

The *Arl13b* transcript (EBI: OTTMUST00000058920.GRCm38) was amplified from mouse cDNA using the primers Arl13b_For and Arl1b_Rev (Table S1) and cloned into pmKate-N2 (Evrogen) as a HindIII/BamHI fragment. Arl13b was then transferred into the plasmid pCAG-H2BCerulean-Fucci2a (R.L.M., unpublished data) as an NheI/AgeI fragment replacing H2B with Arl13b. Subsequently pCAG-Arl13bCerulean-Fucci2a was cut with SalI, blunted and cut with KpnI; pCDNA5/Frt (Life Technologies) was cut with MluI, blunted and cut with KpnI allowing the transfer of CAG-Arl13bCerulean-p2a-Fucci2a as a Blunt/KpnI fragment into pCDNA5/Frt to yield the plasmid pCDNA5-CAG-Arl13bCerulean-Fucci2a. To generate pRosa26-CAG-floxNeo-Arl13bCerulean-Fucci2a, Arl13bCerulean-p2a was PCR amplified using the primers Arl13bCerulean_For and Arl13bCerulean_Rev and cloned as an MluI/BssHII fragment into the single MluI site of the previously described pRosa26-CAG-floxNeo-Fucci2a plasmid (Mort et al., 2014). The transgene was orientated in opposition to the endogenous *Rosa26* promoter to avoid transcriptional interference (Strathdee et al., 2006).

#### Generation of Arl13bCerulean-Fucci2a NIH 3T3 Cells

The Arl13bCerulean-Fucci2a 3T3 cell line was generated using the Flp-In system (Life Technologies). The Neon electroporation system (Life Technologies) was used to co-transfect the cells with pCDNA5-CAG-Arl13bFucci2a and the pOG44 Flp recombinase expressing plasmid (Life Technologies). Cells were trypsinised, washed in PBS and resuspended in Buffer R. 18 μg of pOG44 and 2 μg of pCDNA5-CAG-Arl13bCerulean-Fucci2a were added to each tube. Each tube was then split into 2 × 100 ul electroporations (2 pulses: 1,350 V, 20 ms) and pooled into a single T75 flask containing pre-warmed OPTIMEM (Life Technologies) and incubated overnight. On the second day cells were transferred into DMEM containing, 10% v/v fetal calf serum (FCS), 1% v/v Penicillin/Streptomycin and 100 μg/ml Hygromycin B. After 14 days of Hygromycin B selection the polyclonal cell line was passaged and used for subsequent analyses.

#### Cell Cycle Analysis by FACS

Confirmation of correct cell cycle phase representation by Fucci2 fluorescence was performed by FACS using DAPI intensity as a measure of DNA content. Using a FACS Aria2 SORP cell sorter (Becton Dickinson) cells were sorted into mVenus (488 nm laser, 525/50 nm bandpass filter) and mCherry (560 nm laser, 610/20 nm band pass filter) positive populations. Cells were fixed in 70% ethanol overnight at -20°C. The next day cells were stained with DAPI (5 μg/ml in PBS) and DNA content analysed using the 405 nm laser and 450-50 nm bandpass filter. FACSDiVa Version 6.1 (BD) was used to analyse the data.

#### Arl13bCerulean-Fucci2a NIH 3T3 Cell Migration Assay

In order to determine the orientation of primary cilia during migration, Ar13bCerulean-Fucci2a NIH 3T3 cells were seeded at high density into a custom-made silicon ring mounted on a 24-well glass bottomed plate (Greiner Bio-one) by surface tension. The next day the silicon ring was removed and the media replaced with phenol free DMEM (Millipore) (10% v/v FCS, 1% v/v Penicillin/Streptomycin). Images were taken every 10 mins at a single Z plane. The angle of cilia in relation to the centre of the nucleus was measured in migrating cells after 5 and 10 hours using a custom macro in ImageJ. The angle was then corrected for the orientation of the wound edge with respect to the image. A control experiment was also set up in parallel in which Arl13bCerulean-Fucci2a NIH 3T3 cells were seeded so there was no directional movement and their ciliary angles measured.

#### Mouse Embryonic Stem Cell Targeting

E14Tg2a ES cells were electroporated with linearised pRosa26-CAG-floxNeo-Arl13bCerulean-Fucci2a plasmid using standard procedures. Clones were picked after 14 days of G418 selection. Long range PCR across the 5’ and 3’ homology arms confirmed correct targeting of the endogenous *Rosa26* locus without further genomic rearrangements. Screening across the Rosa26 5′ homology arm (35 cycles: denaturation - 98°C for 10 secs; annealing - 66°C for 10 secs; extension - 72°C for 30 secs) was performed using the primers Xu_Wt_For (Hohenstein et al., 2008) and Rosa5_R1 (Mort et al., 2014) (Figure S1A) to generate a 1.4 kb targeted band. A second control PCR (35 cycles: denaturation - 98°C for 10 secs; annealing - 60°C for 10 secs; extension - 72°C for 30 secs) was conducted to demonstrate DNA integrity using the primers Wt_For and Wt_Rev (Soriano, 1999) to generate a 450 bp wild type band from the Rosa26 locus. Correct targeting was confirmed on the positive clones by PCR amplification (35 cycles: denaturation - 98°C for 10 secs; annealing - 68°C for 10 secs; extension - 72°C for 30 secs) of a 4 kb targeted band across the Rosa26 3′ homology arm using the primers Rosa3_F1 and Rosa3_R2, all primer sequences are outlined in Table S1. All PCR reactions were carried out using 50 ng genomic DNA using Phusion Hotstart II DNA polymerase (Thermo Fisher Scientific) with GC buffer according to the manufacturers standard reaction conditions.

#### Cre-Activation of *R26Arl13b-Fucci2aR* Mouse Embryonic Stem Cell Lines

Correct expression of the Arl13bCerulean-Fucci2a transgene was assessed in targeted mESC clones by transfection with the PGK-Cre plasmid followed by selection of fluorescent clones and confirmation of G418 sensitivity. To excise the neomycin stop cassette and activate Arl13bCerulean-Fucci2a expression, 1 x 10^7^ *R26Arl13b-Fucci2aR* mESCs in 0.5 ml of PBS were combined with 100 μg PGK-Cre plasmid (a kind gift from Dr Laura Lettice, University of Edinburgh) and incubated on ice for 15 minutes. Cells were transferred to an ice-cold 0.4 mm electroporation cuvette and electroporated using the Gene Pulser II (Bio-Rad). Cells were seeded at low density (1000 cells per 10 cm^2^ pre-gelatinised plate), and grown until clones become visible. Fluorescent clones were picked, expanded and checked for G418 sensitivity (250 μg/ml).

#### 2i Conversion of *R26Arl13b-Fucci2a* Mouse Embryonic Stem Cells

2i conversion was achieved by culturing for a minimum of 7 days in 50/50 neurobasal (Gibco) / DMEM/F12 (Gibco) media containing 1 X N2 Supplement (Gibco), 1 X B27 + RA (Gibco), 7.5% w/v BSA (Gibco), 1 X Penicillin/Streptomycin (Corning), 1 μM PD0325901 (Stemgent), 3 μM CHIR99021 (Stemgent), 2mM Glutamine (Gibco), 0.15 mM Monothioglycerol (Sigma) and 1000 U/ml LIF (Esgro). 2i culture conditions are as reported in Ying et al. (2008) with the exception of ESGRO LIF (Millipore) used at 1000 U/ml.

#### Generation of *R26Arl13b-Fucci2aR* Mice

Transgenic mice were produced by blastocyst injection of *R26Arl13b-Fucci2aR* ES cells according to standard methods using C57BL/6J mice. Germline transmission was identified after a single round of ES cell blastocyst injections. Subsequent intercrosses generated *R26Arl13b-Fucci2aR*^*+/+*^, *R26Arl13b-Fucci2aR*^*+/Tg*^ and *R26Arl13b-Fucci2aR*^*Tg/Tg*^ offspring at Mendelian ratios. *R26Arl13b-Fucci2aR* mice were genotyped using the strategy described for *R26Fucci2aR* animals (Mort et al., 2014). Briefly, a duplex PCR reaction (35 cycles: denaturation - 98°C for 10 secs; annealing - 68°C for 10 secs; extension - 72°C for 30 secs) was used with primers R26_Wt_For, R26_Wt_Rev and F2A_Rev (Table S1), reactions were carried out using Phusion Hotstart II DNA polymerase (Thermo Fisher Scientific) with GC buffer according to the manufacturers standard reaction conditions the R26_Wt_Rev primer was used at 50 μM rather than 100 μM. The *R26Arl13b-Fucci2aR* allele was bred to homozygosity and loss of the wild type allele confirmed by PCR to further validate our ES cell screening strategy.

#### Preparation of Primary Ependymal Cultures

To isolate primary ependymal cells, the ventricular zone from E18.5 *R26Arl13b-Fucci2aR*^*+/Tg*^*; CAG-Cre*^*+/Tg*^ embryos was dissected and dissociated by mild trypsinisation in a solution of trypsin/EDTA for 45 minutes followed by pipetting to form a single cell solution. Cells were then seeded onto a collagen-coated glass bottom dish. Cells were cultured in DMEM (10% v/v FCS, 1% v/v Pen/Strep, 1 X GlutMax) until confluent and then serum starved to induce differentiation and the formation of multiple motile cilia.

#### Preparation of Mouse Embryonic Fibroblasts

Mouse embryonic fibroblasts (MEFs) were prepared from individual E12.5 embryos using standard techniques. Briefly, the tail, limbs, head and organs were removed and the remainder of the body transferred to a 6-well plate on ice. In a cell culture hood using sterile techniques the PBS was aspirated off and replaced with 50/50 trypsin/versene. The embryos were then broken up into small pieces using forceps and left for 1 hour, 3 ml of MEF media (Opti-mem (GIBCO), 10% v/v FCS, 1% v/v Penicillin/Streptomycin, 0.1 mM 2-Mercaptoethanol (Sigma)) was added and solution pipetted up and down to dissociate the tissue into single cells and seeded into a T75 tissue culture flask. MEF lines were passaged at least 3 times before being stored in liquid nitrogen in MEF media plus 10% v/v DMSO, 30% v/v FCS. MEF lines were cultured in a humidified 37°C incubator supplied with 5% CO2, maintained at high confluency and split at no greater ratio than 1:3.

#### Bile Duct Isolation and Enrichment

Livers were flushed with phosphate buffered saline through perfusion through the inferior vena cava, then dissected, and digested in DMEM/F12 media containing Collagenase IV (Gibco) and Dispase (Gibco) at 37°C with intermittent agitation until the parenchyma was digested away and the bile ducts appeared visible in the media. Following digestion, bile ducts were collected using a 70 μm filter. Serial PBS washing removed residual cells and resulting bile ducts were used for both live and fixed imaging. For live imaging, bile ducts were suspended in DMEM^gfp^-2 anti-bleaching live cell visualization medium supplemented with rutin (20 mg/L - Evrogen), and NucBlue (Invitrogen) and incubated for 30 minutes at 37°C. Live imaging was performed on an Andor Dragonfly Dual Spinning Disc confocal microscope. For quantitative imaging of cilia length, isolated bile ducts were fixed for 10 minutes in methacarn and permeabilised with PBS + 0.1% Tween20 prior to incubating in protein block for 1 hour. Primary antibodies were allowed to incubate overnight at 4°C and secondary antibodies for two hours at room temperature following PBS washes. Bile ducts were fixed and imaged using confocal microscopy (Nikon A1R+). For organoids, isolated bile ducts were plated with 100% GFR Matrigel (Corning) and cultured in base media (DMEM/F12 with Glutamax, Antibiotic-Antimycotic and HEPES (Gibco)) or growth media (base media supplemented with EGF, HGF, FGF10 (Peprotech), Gastrin, Nicotinamide, N-Acetylcystine, A83-01 (TGFβ inhibitor; Sigma), B-27 (Gibco), Forskolin, Y-27632 (Rock inhibitor; Tocris), and Chir99021 (GSK-3β inhibitor; Miltenyl)).

#### Fluorescence Microscopy and Live Imaging

Fluorescence live cell imaging of cell lines and embryonic tissue was performed on the stage of a Nikon A1R confocal microscope surrounded by an environmental chamber providing 5% CO_2_ in air and maintained at a constant stage top temperature of 37°C. Data was acquired using either a 20x Plan Apochromat VC 0.5 DIC N2, 40x Plan Fluar 0.75 DIC N2 or 60x Plan Apochromat VC 1.2 WI DIC N2 lens through NIS Elements AR software (Nikon Instruments Europe, Netherlands). The scission studies and cilia length comparisons were performed using the multimodal Imaging Platform Dragonfly (Andor Technologies, Belfast, UK) using 20x Plan Apochromat VC 0.75 DIC N2 or air 40x Plan Fluar 0.75 DIC N2. Data were collected in Spinning Disk 25 μm pinhole mode on the high sensitivity iXon888 EMCCD camera. Z stacks were collected using a Mad City Labs Piezo. Data was visualized using IMARIS 8.4 (Bitplane). In our time-lapse dataset, we identified cilia using segmentation and morphological filtering and defined each cilia by a bounding box (the minimum bounding rectangular cuboid that fully encompasses the cilia). The following laser lines and band filters were used; Cerulean – 458 nm, 482/35 (A1R) or 480/40 (Dragonfly); mVenus – 514 nm, 540/30 (A1R) or 620/60 (Dragonfly); mCherry – 561 nm, 595/50 (A1R) or 700/75 (Dragonfly). Cells and tissue were imaged in phenol free DMEM (Millipore) (10% v/v FCS, 1% v/v Penicillin/Streptomycin, 1 X GlutMax) on glass bottomed plates (Greiner Bio-one) except imaging of whole-mount embryos which were imaged in DMEM (50% v/v rat serum, 1% v/vPenicillin/Streptomycin, 1 X GlutaMax) and embryonic lung cultures which were embedded in 50% matrigel:media and mounted on a Lumox membrane (Greiner Bio-one) in a custom built imaging chamber (Mort et al., 2010).

#### Immunofluorescence and Comparison of Cilia Length

To compare cilia length between *R26Arl13b-Fucci2a*^*+/Tg*^ and control mice, tissues were dissected and half maintained in cold PBS for live imaging (see above) while the other half was fixed in 4% w/v PFA/PBS for one hour and cryoprotected though a sucrose/PBS gradient overnight at 4°C before embedding in OCT (Sciegen). Nasal brushings were taken by exposing the nasal septum and scraping cells off the epithelium with an interdental brush (TePe, 0.8 mm ExtraSoft) followed by resuspension in DMEM (isolated from animals at P7-P60). Cells were spread on Superfrost slides, and imaged directly or processed for immunofluorescence. Fixed tissues were embedded in OCT (Scigen, USA) and used for cryo-sectioning. Sections of 12 μm were mounted on Superfrost glass slides, air dried and stored at -80 or used immediately. For immunofluorescence, slides were submerged in PBS to remove excess of mounting media and briefly fixed for 2 mins in 4% w/v PFA/PBS, washed and permeabilised with 0.25% v/v Triton/PBS for 10 minutes. Slides were then washed and blocked in 2-4% w/v BSA, 0.05% v/v Tween-20 in PBS. Primary and secondary antibodies were incubated in the same solution for one hour at room temperature before washing and mounting in Prolong Gold (Invitrogen) for imaging.

#### Image Analysis

Image analysis tasks were performed either on Imaris (Bitplane) or using custom written macros for the Fiji distribution of ImageJ, an open source image analysis package based on NIH Image (Schneider et al., 2012).

### Quantification and Statistical Analysis

Statistical tests were performed using the ‘R’ statistics package, an open source software package based on the ‘S’ programming language (http://www.R-project.org) or with GraphPad Prism. The n value and definition of n along with the statistical test used is indicated in the figure legend of each figure.

## Supplemental Information

Document S1. Figures S1–S7 and Table S1

Document S2. Article plus Supplemental Information
