# Supplementary material for: A Cell/Cilia Cycle Biosensor for Single-Cell Kinetics Reveals Persistence of Cilia after G1/S Transition Is a General Property in Cells and Mice
Source: Dev Cell. 2018 Nov 19;47(4):509–523.e5. doi: 10.1016/j.devcel.2018.10.027 (PMC6251972; doi:10.1016/j.devcel.2018.10.027)
Supplement: Document S1. Figures S1–S7 and Table S1 [file mmc1.pdf]

Developmental Cell, Volume *47*

Supplemental Information

A Cell/Cilia Cycle Biosensor for Single-Cell Kinetics Reveals Persistence of Cilia after G1/S Transition Is a General Property in Cells and Mice

Matthew J. Ford, Patricia L. Yeyati, Girish R. Mali, Margaret A. Keighren, Scott H. Waddell, Heidi K. Mjoseng, Adam T. Douglas, Emma A. Hall, Asako Sakaue-Sawano, Atsushi Miyawaki, Richard R. Meehan, Luke Boulter, Ian J. Jackson, Pleasantine Mill, and Richard L. Mort

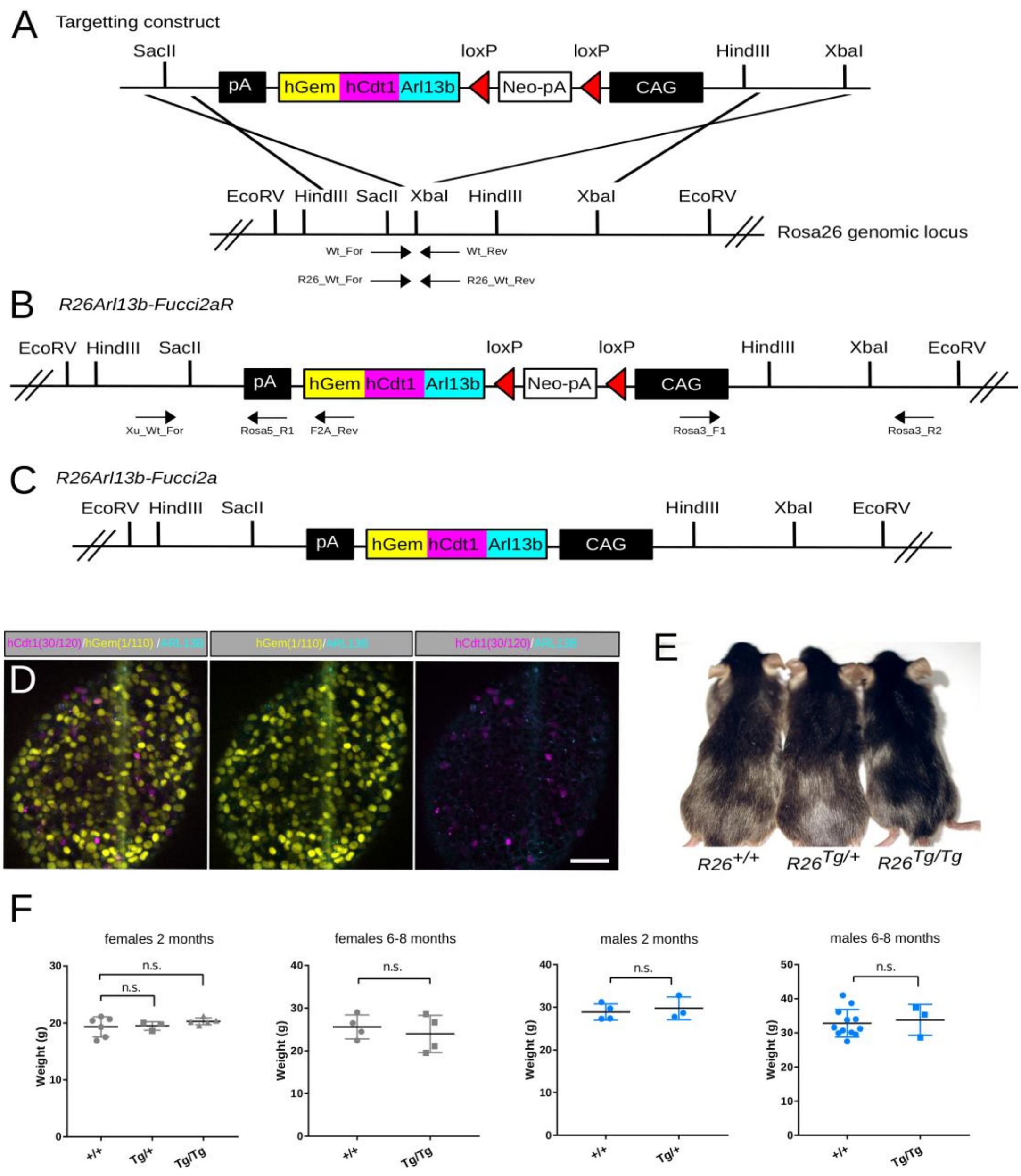

Figure S1, Related to Figure 5. Targeting of Arl13bCerulean-Fucci2a to the *Rosa26* locus in mESCs.

R26Arl13b-Fucci2aR mESCs were generated by homologous recombination of a Cre-recombinase inducible R26Arl13b-Fucci2a targeting construct into the mouse *Rosa26* locus **(A)**. The construct was designed such that expression of the Arl13bCerulean-Fucci2a biosensor is driven by the synthetic CAG promoter and the construct made inducible by inclusion of a loxP flanked neomycin stop cassette between CAG and Arl13bCerulean-Fucci2a sequences. The construct was inserted in the reverse orientation to avoid transcriptional interference between CAG and the endogenous *Rosa26* promoter. **(B)** Successful insertion of the transgene was confirmed by PCR across the 5’ and 3’ homology arms of the

targeting construct using the primer pairs Xu_Wt_For/Rosa5_R1 (5' targeted band), Rosa3_F1/Rosa3_R2 (3' targeted band) and Wt_For/Wt_Rev (wildtype band - See Methods). Genotyping of mice to confirm we could breed the *R26Arl13b-Fucci2aR* allele to homozygosity was performed in a duplex reaction with the primers R26_Wt_For, R26_Wt_Rev and F2A_Rev (See Methods). The inducible allele was termed *R26Arl13b-Fucci2aR.* **(C)** We activated the transgene in ES cells by transfection with PGK-Cre plasmid followed by selection of fluorescent G418-sensitive clones to yield a recombined constitutive *R26Arl13b-Fucci2a* allele. **(D)** Live images of *R26Arl13b-Fucci2a* mESCs cultured under 2i conditions. **(E)** Constitutively expressing *R26Arl13b-Fucci2a* mice were generated by crossing *R26Arl13b-Fucci2aR* mice with *CAG-Cre* mice. *R26Arl13b-Fucci2a* mice were born at mendelian ratios and were phenotypically indistinguishable from their wild type litter mates. No runting or obesity phenotypes associated with ciliopathies were observed. **(F)** We observed no significant difference (Unpaired t tests, $P > 0.5$ in all cases) in the weights of postnatal males and females at 2 months (females, n = 6 wildtype, 3 heterozygotes, 5 homozygotes; males, n = 4 wildtypes and 3 homozygotes), or at 6-8 months (females, n = 4 wildtype and 4 homozygotes; males, n = 12 wildtypes and 3 homozygotes). Scatterplots include; mean and standard deviation, all values shown. Scale bar in D = 100 µm.

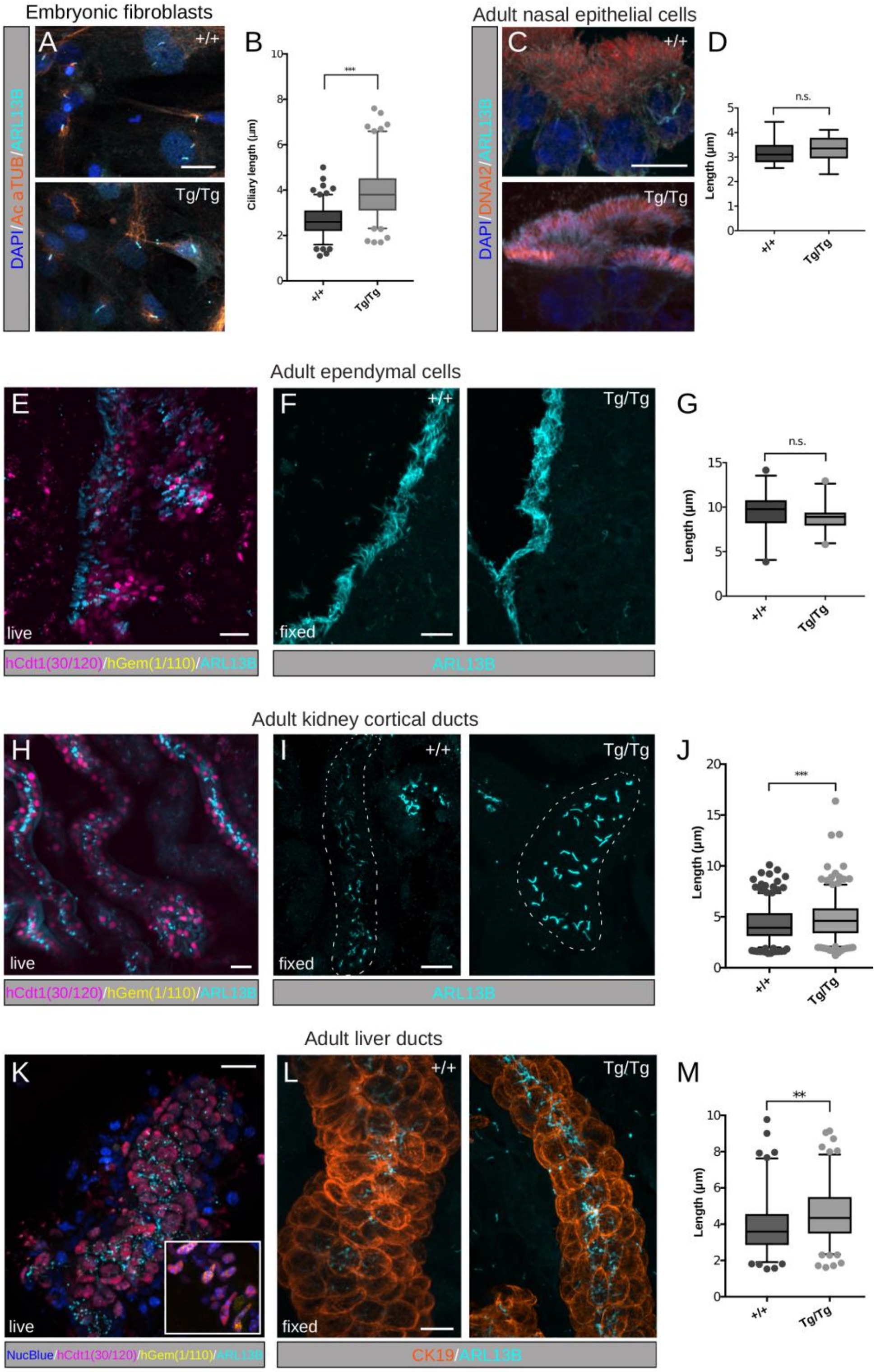

Figure S2, Related to Figure 5. Tissue specific cilia elongation in *R26Arl13b-Fucci2a*$^{Tg/Tg}$ mice. We examined the effect of Arl13b-Cerulean overexpression on cilia length in *R26Arl13b-Fucci2a*$^{Tg/Tg}$ primary cells and adult mice. **(A-B)** Anti-Arl13b staining of primary cilia in *R26Arl13b-Fucci2a*$^{Tg/Tg}$ and wild type mouse embryonic fibroblasts (MEFS) showing that mean cilia length in *R26Arl13b-Fucci2a*$^{Tg/Tg}$ MEFs

(3.955 ± 0.1134 µm, n=121 cilia, n = 1 MEF line, $P < 0.0001$, unpaired t test) is 1.48x greater than in wild type cells (2.675 ± 0.05691 µm, n = 151 cilia, n = 1 MEF line). **(C-D)** Anti-ARL13B (cyan) staining of motile multiciliated (DNAI2: orange) nasal epithelial cells from 6 month old *R26Arl13b-Fucci2a*$^{Tg/Tg}$ (3.33 ± 0.1768 µm, n = 10 cilia, n = 1 animal, $P > 0.05$, unpaired t test) and wild type (3.268 ± 0.2333 µm, n = 7 cilia, n = 1 animal) mice showing no difference in cilia length. **(E-G)** ARL13B localisation (cyan) in the adult (aged 6 months) brain at the 3rd ventricle showing live expression of the biosensor by vibratome (E), or fixed immunofluorescence of the ependymal cells (F) between *R26Arl13b-Fucci2a*$^{Tg/Tg}$ and wild type mice. Quantitation of length of ARL13B+ cilia (G) revealed no significant difference between *R26Arl13b-Fucci2a*$^{Tg/Tg}$ (8.881 ± 0.338 µm, n = 24 cilia, n = 4 animals, $P > 0.05$, unpaired t test) and wild type mice (9.414 ± 0.471 µm, n = 27 cilia, n = 4 animals). **(H-J)** ARL13B localisation (cyan) in the adult kidney cortex (aged 2 months) showing live expression from the biosensor by vibratome (H), or fixed immunofluorescence of the cortical ductal cells (I) between *R26Arl13b-Fucci2a*$^{Tg/Tg}$ and wild type mice. Regardless of genotypes ducts showed cilia of variable lengths and intensities such that analysis was confined to ducts (dashed area) with segmentable cilia, shown (J). A significant 1.13 fold increase in primary cilia length was observed between cortical ducts of *R26Arl13b-Fucci2a*$^{Tg/Tg}$ mice (4.789 ± 0.1186 µm, n = 281 cilia, n = 4 animals, $P < 0.001$, unpaired t test) and wild type (4.251 ± 0.09109 µm, n = 338 cilia, n = 4 animals). **(K-M)** ARL13B localisation (cyan) in bile ducts isolated from adult (aged 4 months) livers showing live expression from the biosensor (K), or fixed whole-mount immunofluorescence where cholangiocytes (CK19: orange) possess long cilia (L). Quantification of ARL13B+ cilia length (M) on cholangiocytes reveals a significant 1.25x increase in length between *R26Arl13b-Fucci2a*$^{Tg/Tg}$ (4.601 ± 0.1337 µm, n = 156 cilia, n = 3 mice, $P < 0.05$, unpaired t test) and wild type mice (3.975 ± 0.1542 µm, n = 119 cilia, n = 1 animal). Boxplots indicate; 5th percentile, 95th percentile, median and interquartile range, outliers are shown. Scale bar in A = 20 µm, scale bar in C = 10 µm, scale bars in E,F,H,I, and L = 20 µm.

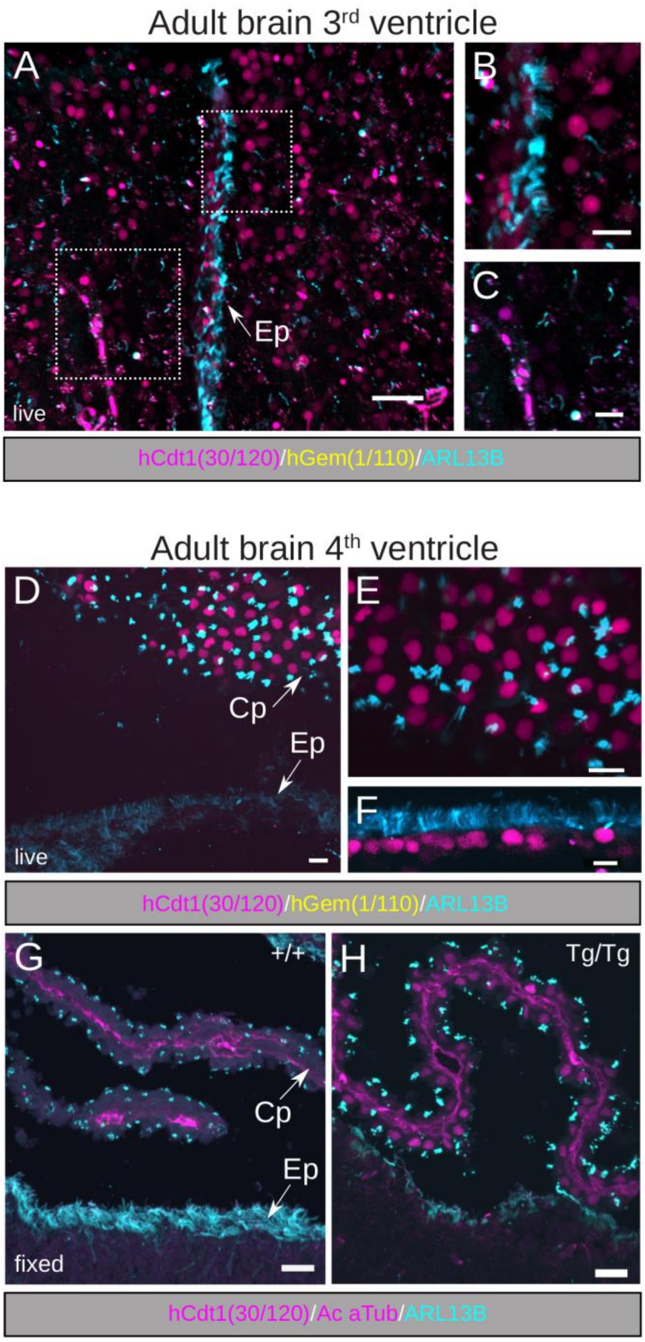

Figure S3, Related to Figure 5. Arl13b-Cerulean sensitively labels diverse cilia types in *R26Arl13b-Fucci2a* mice. Given the challenges of labelling cilia in distinct cell types, especially in the brain, we examined the localisation of ARL13B-Cerulean in live sections from adult brains of *R26Arl13b-Fucci2a*$^{Tg/Tg}$ mice. **(A-C)** ARL13B+ cilia are detected throughout the 3rd ventricle of a *R26Arl13b-Fucci2a*$^{Tg/Tg}$ mouse (aged 6 months). In contrast to the embryonic samples analysed, the majority of cells in adult brain were post-mitotic residing in G0/G1 - labelled with mCherry-hCdt1(30/120). Tufts of ARL13B+ multiciliated ependymal cells (A: Ep and magnified in B) and single primary cilia of neurons (A and magnified in C) are

robustly labelled with ARL13B-Cerulean. **(D-H)** Live imaging in a single field in the adult 4th ventricle (aged 2-months) revealed ARL13B+ cilia on neurons (not shown), as well as motile, multiciliated ependymal cells (D: Ep and F) and in the highly modified, multiciliated cells of the choroid plexus (D: Cp and E) of *R26Arl13b-Fucci2a*$^{Tg/Tg}$ animals (n = 3). **(G-H)** Fixed immunofluorescence with antibodies to ARL13B (cyan) and acetylated α tubulin (magenta) reveal that unlike control samples, *R26Arl13b-Fucci2a*$^{Tg/Tg}$ cilia are very brightly labelled. Note that the choroid plexus is saturated in G despite the image being acquired at a lower exposure than the control sample (H). Ep = ependymal, Cp = choroid plexus, scale bar in A = 50 µm, scale bars in B, C, E, G, H = 20 µm, scale bars in D = 30 µm, and F = 10 µm.

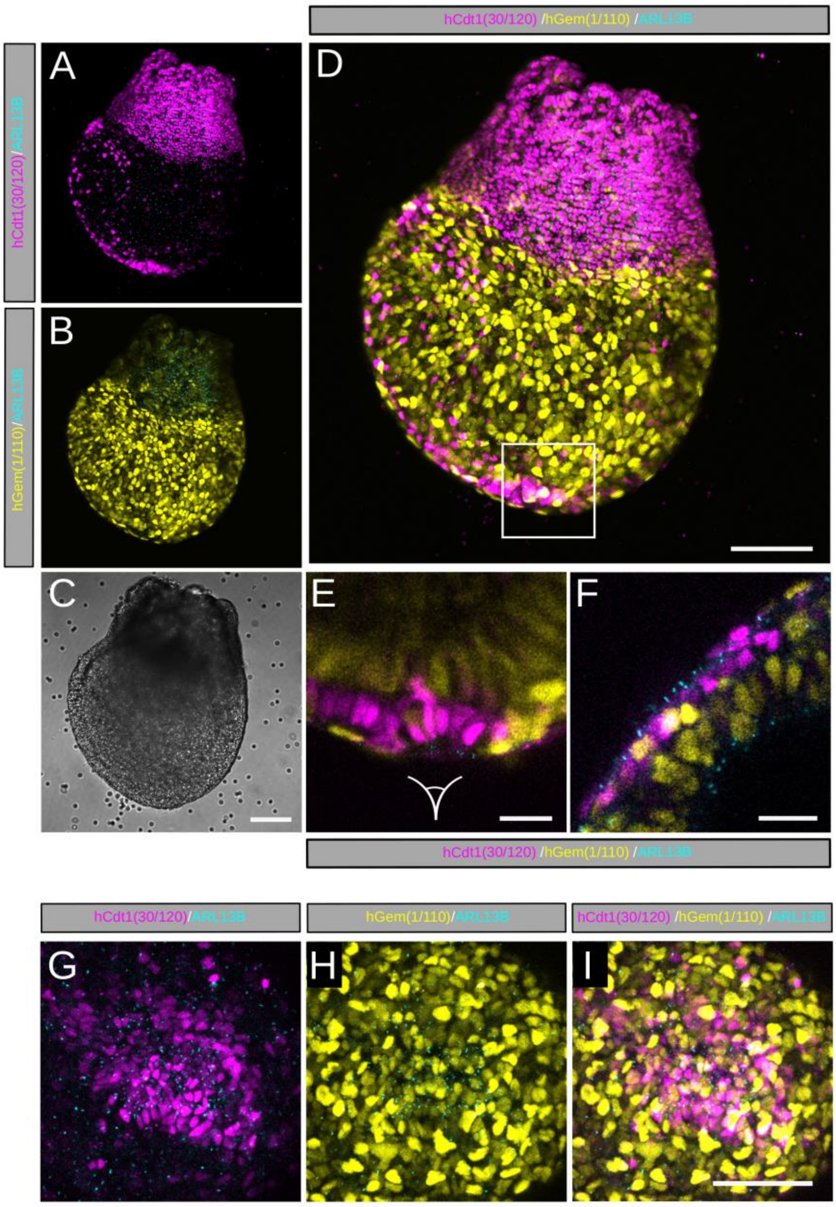

Figure S4, related to Figure 5. The node in E7.5 *R26-Arl13b-Fucci2aR$^{+/Tg}$; CAG-Cre^{+ve}* embryos contains a ciliated mCherry-hCdt1 positive population. Ubiquitous Arl13bCerulean-Fucci2a expression was achieved by crossing *R26-Arl13b-Fucci2aR* mice with ubiquitous *CAG-Cre* mice. Confocal imaging was performed on live immobilised E7.5 embryos. **(A-D)** A Z-projection of an e7.5 *R26-Arl13b-Fucci2aR$^{+/Tg}$; CAG-Cre^{+ve}* embryo. In all cases (n = 4, *R26Arl13b-Fucci2a$^{+/Tg}$* E7.5 embryos from 3 litters) the extraembryonic ectoderm (proximal) lineages are predominantly non-proliferative (mCherry-hCdt1(30/120) dominates - A) while the embryonic visceral endoderm and epiblast lineages are highly proliferative (mVenus-hGem(1/110) dominates - B). The majority of cells in the embryonic lineages are

actively cycling in S/G2/M and therefore labelled with mVenus-hGem(1/110), however a collection of mCherry-hCdt1(30/120) positive cells in G1/G0 is evident at the anterior pole of the embryo consistent with the node (box in D). **(E-F)** Single planes of the box in D clearly showing the node as a ciliated population of mCherry-hCdt1(1//110) positive cells projecting into a concave compartment. **(G-I)** Imaging of the node from the anterior perspective (indicated by the eye in E). Scale bar in C-D = 100 µm, scale bars in E = 50 µm, scale bars in G-I = 100 µm.

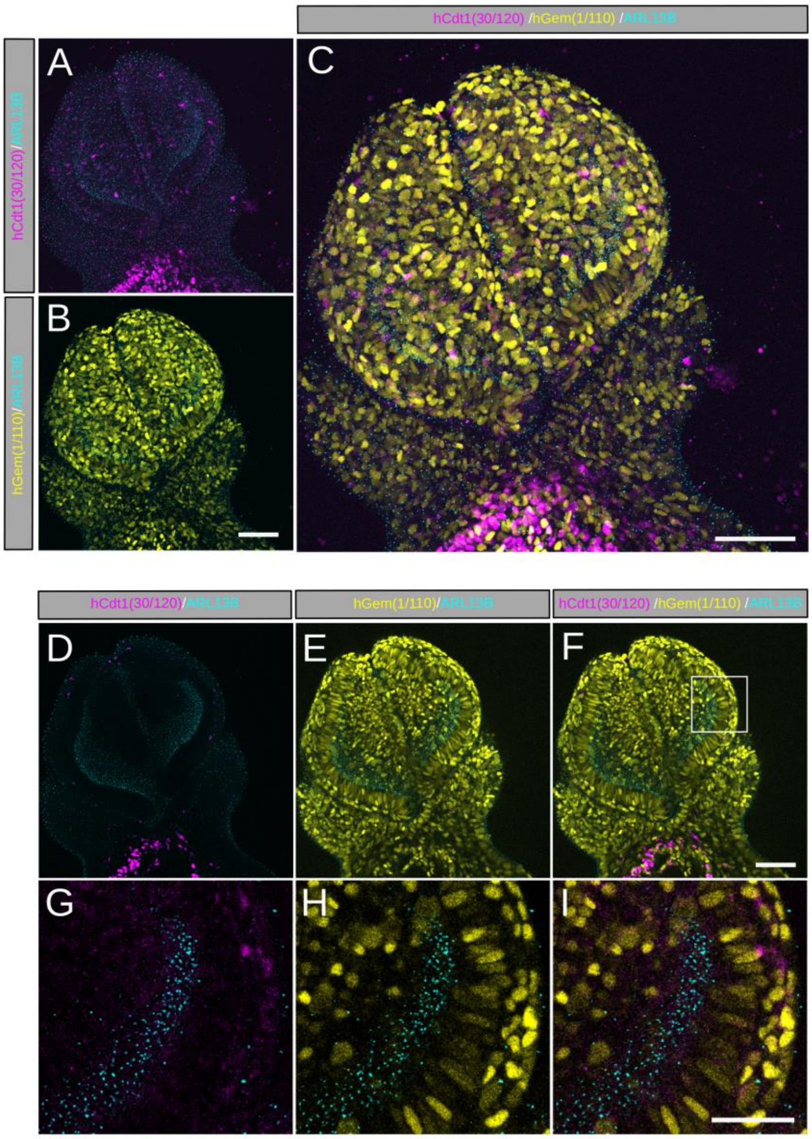

Figure S5, related to Figure 5. Primary cilia are located on the luminal surface of cells lining the lateral ventricles before neurogenesis. (A-C) A Z-projection of an E8.5 prosencephalon from a *R26Arl13b-Fucci2aR*$^{+/Tg}$*; CAG-Cre*$^{+ve}$ embryo. In all cases (n = 8, *R26Arl13b-Fucci2a*$^{+/Tg}$ E8.5 embryos from 2 litters) the majority of cells lining and within the future forebrain were identified in S/G2/M phases of the cell cycle labelled with mVenus-hGem(30/120), a large proportion of cells were ciliated regardless of cell cycle stage. **(D-F)** Confocal optical sectioning approximately 40 μm into the embryo revealed ventricles surrounded by the perpendicularly orientated pseudostratified neuroepithelium. **(G-I)** Magnification of ventricular lumen showing a high density of ARL13B-Cerulean labelled cilia projecting into the luminal space. Scale bars in B, C and F = 100 μm, scale bar in I = 50 μm.

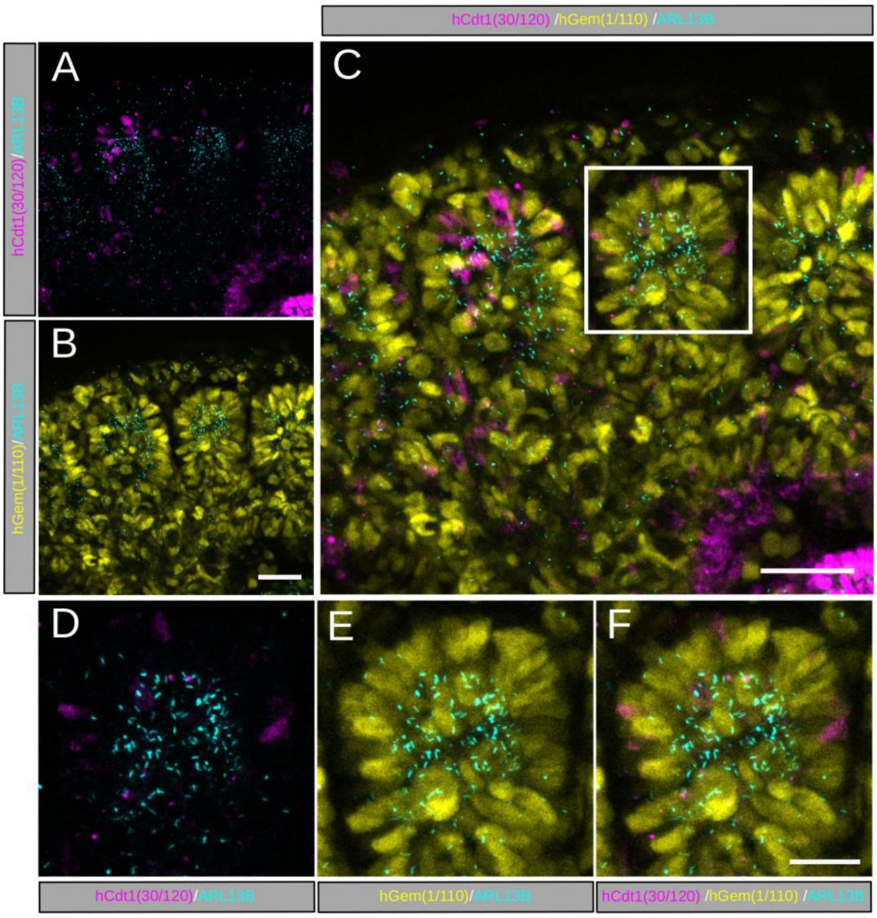

Figure S6, Related to Figure 5. Somites are highly proliferative and contain a high density of primary cilia. (A-C) In all cases (n = 8, *R26Arl13b-Fucci2a*$^{+/Tg}$ embryos from 2 litters) within the cervical region of E8.5 (5-8 somite stage) *R26-Arl13b-Fucci2aR*$^{+/Tg}$*; CAG-Cre*$^{+ve}$ embryos, somites were recognisable as segmented clusters of proliferating cells predominantly in S/G2/M phases of the cell cycle. **(D-F)** A magnification of a single somite highlights a high density of primary cilia within each somite core. Scale bars in B, C = 100 µm, scale bar in F = 25 µm.

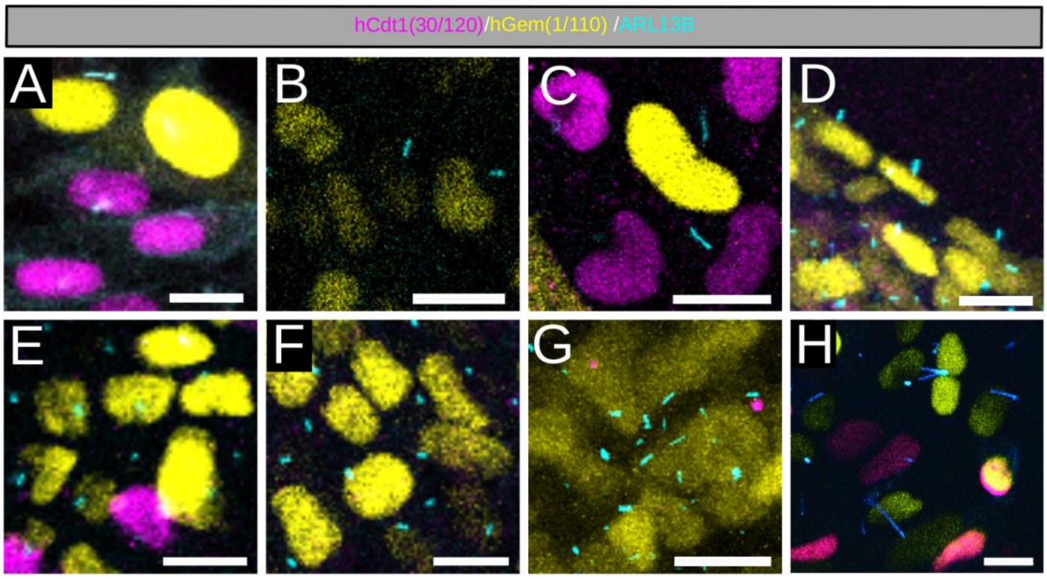

Figure S7, related to Figure 5. Primary cilia are present during S/G2/M phases of the cell cycle *in vitro* and *in vivo*. Confocal images of cells expressing the Arl13bCerulean-Fucci2a transgene indicating ARL13B+ primary cilia located on cells in S/G2/M phases of the cell cycle (yellow - mVenus-hGem(1/110) positive): **(A)** NIH 3T3 cells, **(B)** mouse embryonic stem cells, **(C)** primary ependymal cells, **(D)** the surface of an E8.5 embryo, **(E)** the epiblast of an E7.5 embryo, **(F)** an E8.5 forebrain, **(G)** the centre of an E8.5 somite and **(H)** an adult hepatic ductal organoid. All scale bars = 15 µm.

Table S1, related to STAR Methods: Oligonucleotide sequences used

**Name**	**Sequence (5' - 3')**
**Oligonucleotide sequences used for cloning (restriction endonuclease sites are underlined):**	
Arl13b_For	CGGCAC<u>AAGCTT</u>ATGTTCAGTCTGATGGCCAACTG
Arl13b_Rev	GCCGTC<u>GGATCC</u>CCTGAGATCGTGTCCTGAGCATC
Arl13bCerulean_For	CGGCAC<u>ACGCGT</u>ATGTTCAGTCTGATGGCCAACTG
Arl13bCerulean_Rev	GCCGTC<u>GCGCGC</u>AGGTCCAGGGTTCTCCTCCACGTCTCCAGCCTGCTTCAGCAG
**Oligonucleotide sequences used for screening ES cells:**	
Wt_For	AAAGTCGCTCTGAGTTGTTAT
Wt_Rev	GGAGCGGGAGAAATGGATATG
Xu_Wt_For	GGCGGACTGGCGGGACTA
Rosa5_R1	CCGTAAATAGTCCACCCATTGACG
Rosa3_F1	GGTGGGCTCTATGGCTTCTG
Rosa3_R2	GGAGTAGTTACTCCACTTTCAAG
**Oligonucleotide sequences used for genotyping mice (Wt = 604 bp, Mutant = 880 bp):**	
R26_Wt_For	CAAAGTCGCTCTGAGTTGTTATCAG
R26_Wt_Rev	GGAGCGGGAGAAATGGATATGAAG
F2A_Rev	TGGCGGCCGCTCGAGATGAATC
